# *Feroxichthys yunnanensis* gen. et sp. nov. (Colobodontidae, Neopterygii), a large durophagous predator from the Middle Triassic (Anisian) Luoping Biota, eastern Yunnan, China

**DOI:** 10.7717/peerj.10229

**Published:** 2020-10-20

**Authors:** Guang-Hui Xu

**Affiliations:** 1 Key Laboratory of Vertebrate Evolution and Human Origins of Chinese Academy of Sciences, Institute of Vertebrate Paleontology and Paleoanthropology, Chinese Academy of Sciences, Beijing, China; 2 CAS Center for Excellence in Life and Paleoenvironment, Beijing, China

**Keywords:** Osteology, Phylogeny, Colobodontidae, Neopterygii, Actinopterygii

## Abstract

Neopterygii is a large group of ray-finned fishes which underwent a rapid radiation in the Middle Triassic. Until recently, 11 stem neopterygians have been recovered from the early Middle Triassic Luoping Biota in eastern Yunnan, China, and they are small to medium-sized fishes. Here, I report the discovery of a new stem neopterygian, *Feroxichthys* y*unnanensis* gen. et sp. nov. from the Luoping Biota, which represents the first evidence of large-sized stem neopteygians in this biota with a total length of ~340 mm (290 mm in standard length). The skull of the new taxon is exceptionally well-preserved, showing some peculiar features rarely known in other stem neopterygians, for example fusion of paired premaxillae, fusion of lacrimal with maxilla, and a fused parieto-dermopterotic with a strong posterior process. Phylogenetic studies recover *Feroxichthys* as a basal colobodontid, and a revised diagnosis of this family is presented. The feeding apparatus indicates that *Feroxichthys* might have been predominantly durophagous, resembling other colobodontids. However, the anterior peg-like teeth in the jaws of *Feroxichthys* are much longer and stronger than other colobodontids, enabling a more powerful initial prey capture before food was passed posteriorly to molariform teeth for crushing in the oral cavity. As a mysterious large durophagous predator previously unknown from the Luoping Biota, the new finding is important not only for understanding the early diversification of neopterygians during this age but also for investigating the trophic structure in this marine ecosystem.

## Introduction

Neopterygii is a diverse group of ray-finned fishes, including Teleostei, Holostei (e.g. gars and bownfin), and closely related fossil taxa (Regan, 1923; Brough, 1931, 1939; Stensiö, 1932; Lehman, 1952; Schaeffer, 1956; Patterson, 1973, 1982; Gardiner, Maisey & Littlewood, 1996; Grande & Bemis, 1998; Arratia, 1999; Sallan, 2014; Friedman, 2015; López-Arbarello & Sferco, 2018; Clarke & Friedman, 2018; Xu & Ma, 2018; Xu, 2019). Teleostei, the largest subgroup of neopterygians or even vertebrates today, has no fossil record until the late Ladinian (~240 Ma), late Middle Triassic (Arratia, 2013, 2015; Tintori et al., 2015). In the early Middle Triassic (Anisian), members of the Neopterygii are mainly represented by stem neopterygians and holosteans (Hurley et al., 2007; Cavin, 2010; Grande, 2010; Romano et al., 2016; Xu et al., 2019). The Triassic stem neopterygians, traditionally grouped in the paraphyletic ‘Subholostei’, have long attracted the attention of palaeoichthyologists interested in the early diversification of this clade (Bürgin, 1992; Lombardo, 2001; Mutter, 2002, 2004; Sun et al., 2009; Xu, Gao & Coates, 2015; Xu, Zhao & Shen, 2015; Xu, Ma & Zhao, 2018; Wen et al., 2019; Xu, 2020a).

The family Colobodontidae is a group of large-sized marine stem neopterygian fishes (up to 650 mm in total length) with a durophagous feeding adaption (Stensiö, 1921; Bürgin, 1996; Mutter, 2002, 2004; Sun et al., 2008; Cartanyà et al., 2015). As previously restricted by Mutter (2002, 2004), the family includes two genera *Colobodus* and *Crenilepis*; although another genus, ‘*Ticinocolobodus*’ (Mutter, 2002) was once proposed, it has not been formally published and its anatomical features (represented by a single incomplete specimen) are not well-known. *Colobodus* includes at least five species from the Middle to Late Triassic in Europe and South China, and *Crenilepis* is represented by the type species *C. sandbergeri* from the Middle Triassic of Germany, Italy and Switzerland in Europe (Mutter, 2002, 2004; Sun et al., 2008; Cartanyà et al., 2015; Li et al., 2019). The Colobodontidae has long been referred to the ‘Perleidiformes’ (probably paraphyletic; Gardiner & Schaeffer, 1989; Mutter, 2002; Cartanyà et al., 2015; Xu, Gao & Coates, 2015; Xu, Zhao & Shen, 2015; Xu, 2020a), but it has rarely been included in phylogenetic analyses regarding the relationships of early neopterygians.

In the past decade, 11 stem neopterygian species (in 11 genera) were recovered from the early Middle Triassic Luoping Biota or Lagerstätte in eastern Yunnan, China (Lombardo et al., 2011; Geng et al., 2012; Sun et al., 2009, 2012, 2015; Lin et al., 2011; Xu & Ma, 2016; Xu & Zhao, 2016; Wen et al., 2019; Xu, 2020a). Among them, the peltopleurids, habroichthyids, venusichthyids, platysiagids and basal louwoichthyids are small-sized fishes with a standard length (SL) of 30–50 mm, and the rest (*Luopingichthys*, *Fuyuanperleidus* and several ‘perleidids’) medium-sized with a SL of 85–190 mm. At the order level, these stem neopterygians are taxonomically referred to ‘Perleidiformes’, Peltopleuriformes (sensu Xu & Ma, 2016), Platysiagiformes and Louwoichthyiformes (Xu, 2020a), in addition to few without to a particular order within this clade.

Here, I report the discovery of a new stem neopterygian on the basis of a specimen collected in 2010 from the middle part of the Second (Upper) Member of the Guanling Formation exposed in Luoping, eastern Yunnan. The fossil fish preserved in a large slab (540 mm × 460 mm) of micritic limestone is fully exposed after four months’ preparation. It is nearly complete (although part of fins and a small region of flank scales are missing) with a total length of about 340 mm (SL = 290 mm), representing the largest known stem neopterygian from the Luoping Biota. Impressively, the skull is exceptionally well-preserved, possessing some derived features of the Colobodontidae (Mutter, 2004; Sun et al., 2008; Cartanyà et al., 2015). Meanwhile, it exhibits peculiar fusions of some cranial bones unknown in other colobodontids or even rarely seen in other stem neopterygians. No additional specimens of this stem neopterygian were collected in the last decade. Based on the type and only known specimen, I presented the new taxon and incorporated it into a phylogenetic analysis to illuminate its relationships with other stem neopterygians in this article.

The early Middle Triassic Luoping Biota is a Lagerstätte renowned by its exceptional preservation and taxonomic richness (including abundant invertebrates, fishes, marine reptiles and plants; Zhang et al., 2009; López-Arbarello et al., 2011; Wu et al., 2011; Xu & Wu, 2012; Feldmann et al., 2012; Huang et al., 2013; Xu, Zhao & Coates, 2014; Xu & Ma, 2016; Xu & Zhao, 2016; Wen et al., 2019; Xu et al., 2019; Xu, 2020a; see review of Benton et al., 2013). The age of this Lagerstätte (Pelsonian, Anisian, ~244 Ma) is well constrained by conodont studies (Zhang et al., 2009), and consequently it provides a unique window into the recovery and radiation of Triassic ecosystems ~8 Myr after the end-Permian mass extinction. The fossil beds are composed of thinly laminated micritic limestones alternating with silty limestones, indicating a semi-enclosed intraplatform depositional environment in the early Middle Triassic Yangtze Sea, a part of the eastern Palaeotethys Ocean (Hu et al., 2011; Metcalfe, 2011; Benton et al., 2013).

## Materials and Methods

The specimen is curated at the fossil collections of the Institute of Vertebrate Paleontology and Paleoanthropology (IVPP), Chinese Academy of Sciences in Beijing, China. It was prepared by air-chisels, accompanied with sharp steel needles. For better contrast, the specimen was dusted with ammonium chloride (NH_4_Cl) before being photographed. Because the sensory pores are very small and hard to identify in the strongly ornamented cranial bones, and the medial teeth in the oral cavity are not exposed in the specimen, X-ray scanning technology was used to reveal these anatomical features. This scanning was carried out using a micro-computed laminography system at the Key Laboratory of Vertebrate Evolution and Human Origins of Chinese Academy of Sciences. The relative position of fins and scale counts were expressed following Westoll (1944). The traditional actinopterygian nomenclatures (Gardiner & Schaeffer, 1989; Bürgin, 1992; Grande & Bemis, 1998) are generally followed, for ease of comparison with most existing literature. The segmented and unbranched rays anterior to the principal rays of the fins are termed as procurrent rays, and the rudimentary ray is confined to the segmented (unsegmented, occasionally) and unbranched ray with an abbreviated or rudimentary base (which does not reach the anterior tip of the base of the first principal ray) in the caudal fin, following the nomenclature promoted by Arratia (2008, 2009).

The phylogenetic framework for the discussions provided herein is based on the results of a phylogenetic analysis including 130 morphological characters and 53 actinopterygian taxa. The characters were mainly adopted from Xu (2020a), which in turn were derived from other analyses of actinopterygian phylogeny (Gardiner & Schaeffer, 1989; Gardiner, Maisey & Littlewood, 1996; Gardiner, Schaeffer & Masserie, 2005; Grande & Bemis, 1998; Arratia, 1999, 2013; Coates, 1999; Cloutier & Arratia, 2004; López-Arbarello & Zavattieri, 2008; Grande, 2010; Xu & Gao, 2011; Xu et al., 2012; Xu, Gao & Finarelli, 2014; Xu, Zhao & Coates, 2014; Xu, Gao & Coates, 2015; Xu, Zhao & Shen, 2015; Xu, Ma & Zhao, 2018). All characters were unordered and equally weighted. In addition to the new taxon presented here and all 44 taxa in the recent analysis of Xu (2020a), eight newly added taxa are *Colobodus bassanii*, *C. baii*, *C. giganteus*, *Crenilepis sandbergeri*, *Luganoia lepidosteoides*, *L. fortuna*, *Peltoperleidus ducanensis* and *P. macrodontus* (see Supplemental Material). Thus, the sampled taxa include most *Perleidus*-like (‘perleidiform’) neopterygians based on well-preserved specimens. Because the focus of this analysis is on the interrelationships of early neopterygian clades (especially those traditionally grouped in the paraphyletic grades ‘Subholostei’ or ‘Perleidiformes’), the Cladistia and many other basal actinopterygians are not included. The data matrix was generated by WinClada 1.00.08 (Nixon, 2002). Tree searches were accomplished with the heuristic search algorithm (gaps treated as missing data; 1,000 random addition sequence replicates; tree bisection-reconnection (TBR) branch-swapping, with 10 trees held at each step and multiple trees saved) in PAUP* 4.0b10 (Swofford, 2003). The basal actinopterygian *Moythomasia durgaringa* (Gardiner, 1984) was selected as the out-group taxon, following previous hypotheses of early actinopterygian phylogeny (Gardiner & Schaeffer, 1989; Coates, 1999; Gardiner, Schaeffer & Masserie, 2005; Xu & Gao, 2011; Xu, Gao & Finarelli, 2014; Giles et al., 2017).

The electronic version of this article in Portable Document Format (PDF) will represent a published work according to the International Commission on Zoological Nomenclature (ICZN), and hence the new names contained in the electronic version are effectively published under that Code from the electronic edition alone. This published work and the nomenclatural acts it contains have been registered in ZooBank, the online registration system for the ICZN. The ZooBank Life Science Identifiers (LSIDs) can be resolved and the associated information viewed through any standard web browser by appending the LSID to the prefix http://zoobank.org/. The LSID for this publication is: urn:lsid:zoobank.org:pub:868619C7-6001-4BCE-A2B9-6C3CC7192CA5. The online version of this work is archived and available from the following digital repositories: PeerJ, PubMed Central and CLOCKSS.

## Results

### Systematic Paleontology

Actinopterygii Cope, 1887

Neopterygii Regan, 1923

Colobodontidae Andersson, 1916

**Emended diagnosis.** A family of stem neopterygian fishes distinguished from other members of this clade by the following unique combination of features (those unique among early neopterygians identified with an asterisk): anterior portions of frontal bones partly separated by broad rostral or postrostral bone; presence of multiple supraorbitals arranged in more than one horizontal row (single row in some derived forms); absence of suborbital; opercle larger than subopercle; subopercle with deep anterodorsal process (*); rounded molariform teeth on coronoids, prearticular and pterygoids; seven to eighteen pairs of branchiostegal rays; three to nine segmented procurrent rays in dorsal lobe of caudal fin; and caudal rays ornamented with rounded or elongated ganoid tubercles (*).

Content: *Colobodus* Agassiz, 1844; *Crenilepis* Dames, 1888; and *Feroxichthys* gen. nov.

Type genus: *Colobodus* Agassiz, 1844

Geographical distribution and age: Baden-Württemberg, Germany; Monte San Giorgio, Switzerland; Perledo, Italy; Catalonia, Spain; Yunnan and Guizhou, China; Pelsonian (Anisian) to Carnian, Middle to Late Triassic.

*Feroxichthys* gen. nov.

LSID urn:lsid:zoobank.org:act:3E8B9492-862D-458D-9EF8-9C68F6C340B1

**Etymology.** The Latin epithet ‘ferox’ means ferocious, and the Greek suffix ‘-ichthys’ means fish.

**Type species.** *Feroxichthys yunnanensis* gen. et sp. nov.

**Diagnosis.** Same as for the type and only known species.

*Feroxichthys yunnanensis* gen. et sp. nov.

LSID urn:lsid:zoobank.org:act:F4ED5C97-1601-41D8-A520-EF2CBB4AF4F5

(Figs. 1–5)

**Figure 1:**
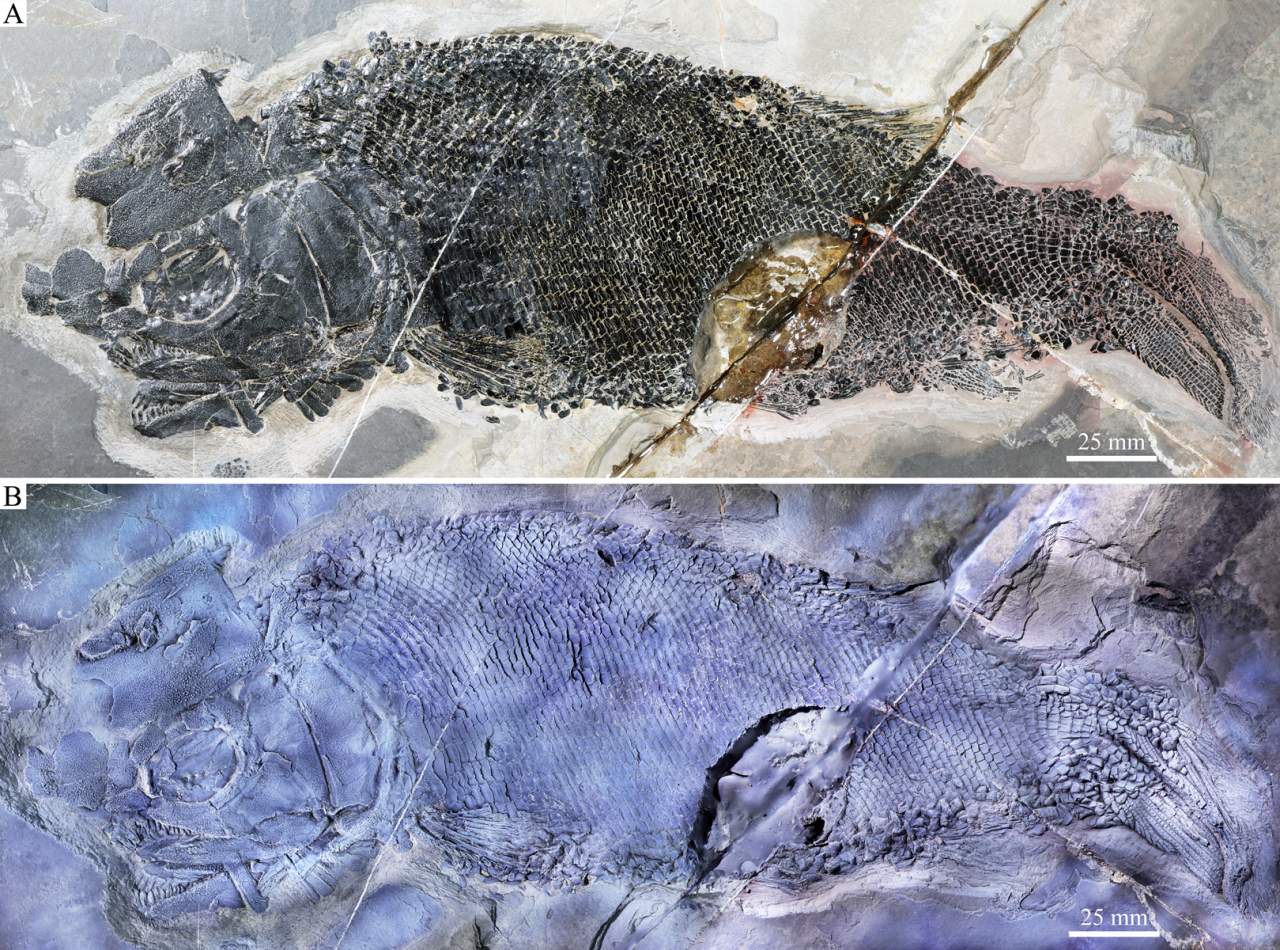
Entire specimen of holotype. Entire specimen of *Feroxichthys yunnanensis* gen. et sp. nov., IVPP V25692 (holotype). (A) Original specimen. (B) Specimen dusted with ammonium chloride.

**Figure 2:**
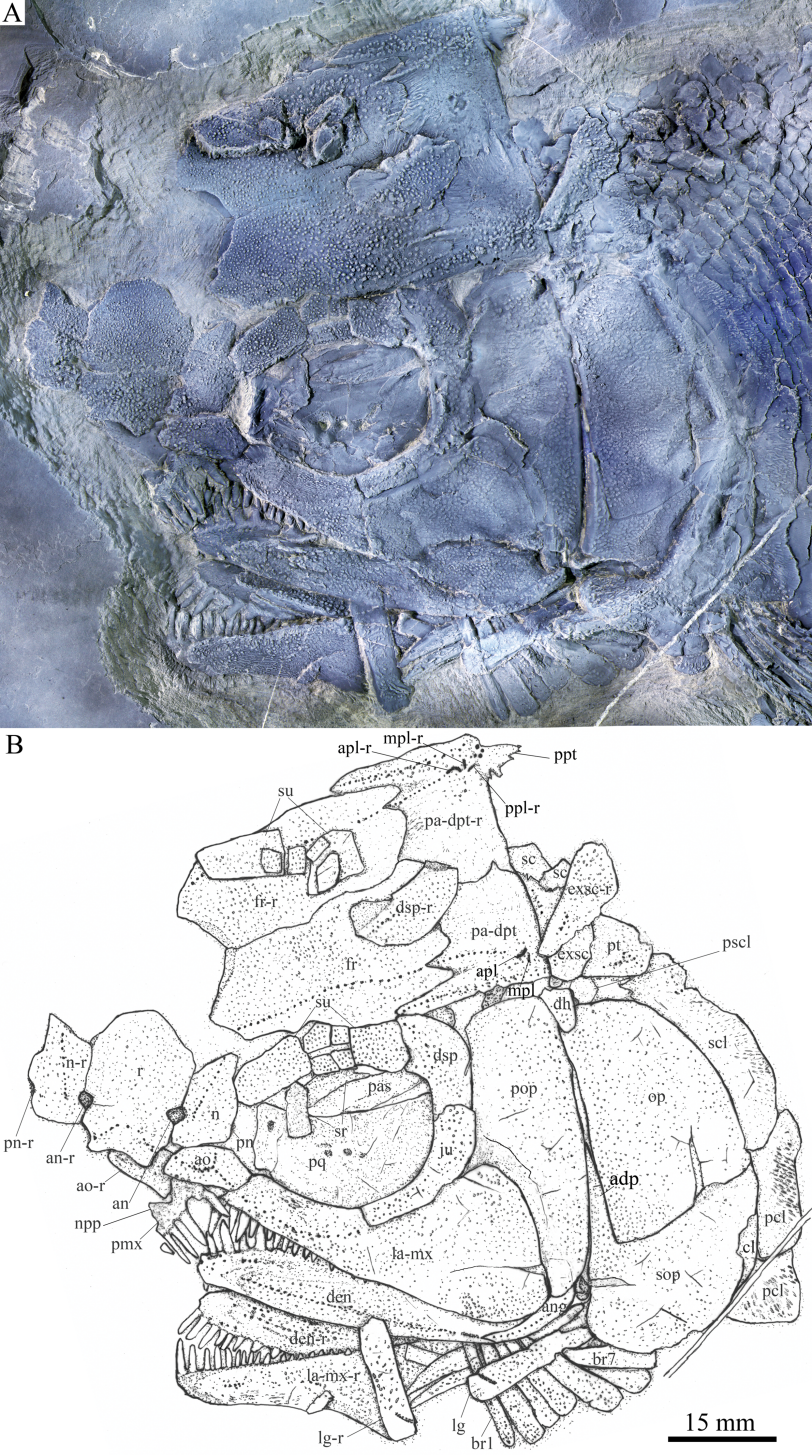
Skull and pectoral girdle of the holotype. Skull and pectoral girdle of *Feroxichthys yunnanensis* gen. et sp. nov., IVPP V25692 (holotype). (A) Photograph. (B) Line-drawing.

**Figure 3:**
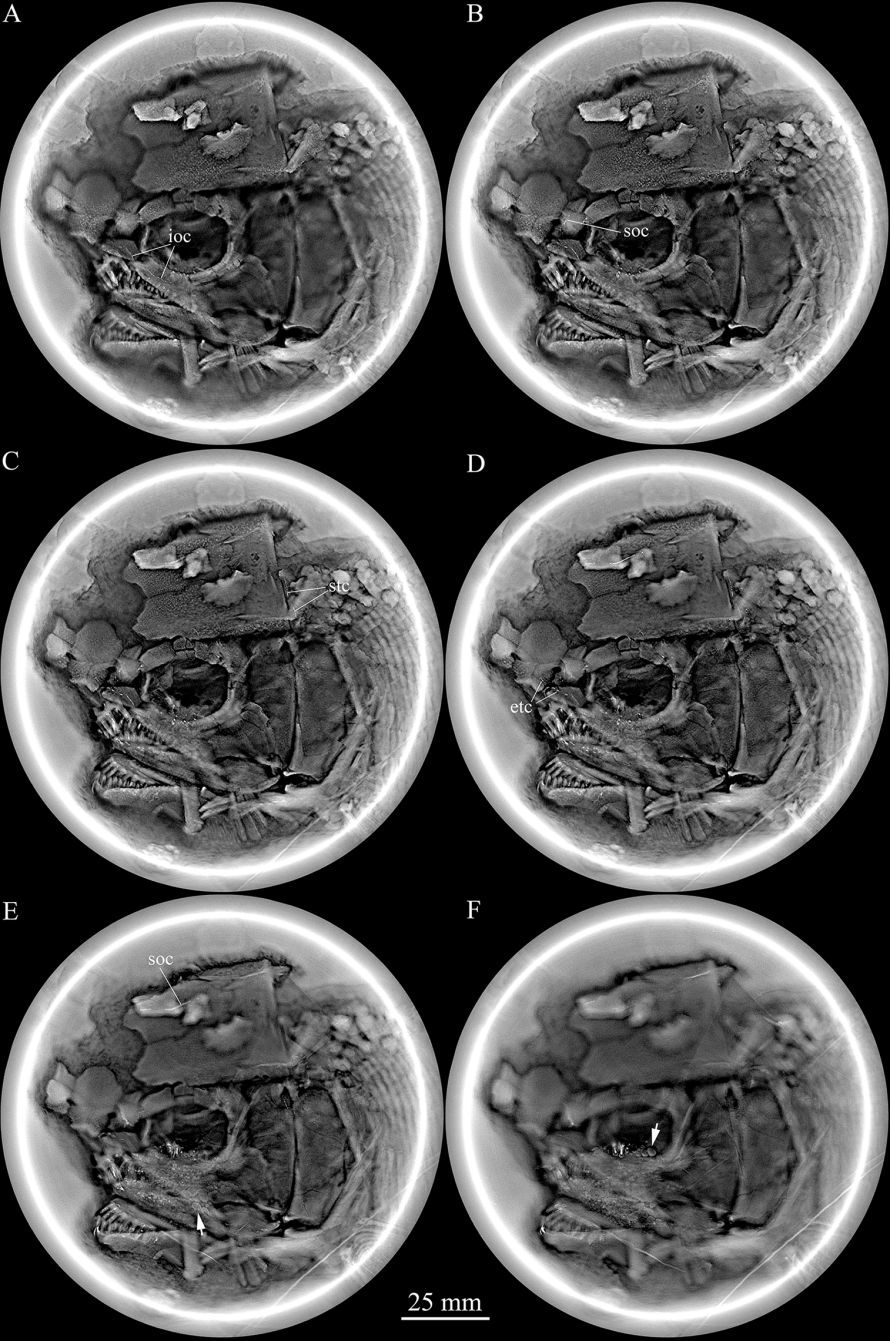
Micro-computed scanning slices of cranial bones. Selected micro-computed scanning slices of cranial bones of *Feroxichthys yunnanensis* gen. et sp. nov., V25692 (holotype). Noting that the infraorbital canal extending from the antorbital into the lacrimo-maxilla (A), the supraorbital canal in the nasal (B), the supratemoral commissure in the extrascapular (C), the ethmoid commissure extending from the antorbital to the rostral (D), the supraorbital canal in the frontal and molariform teeth on the prearticular (E) and molariform teeth on the pterygoid bones (F).

**Figure 4:**
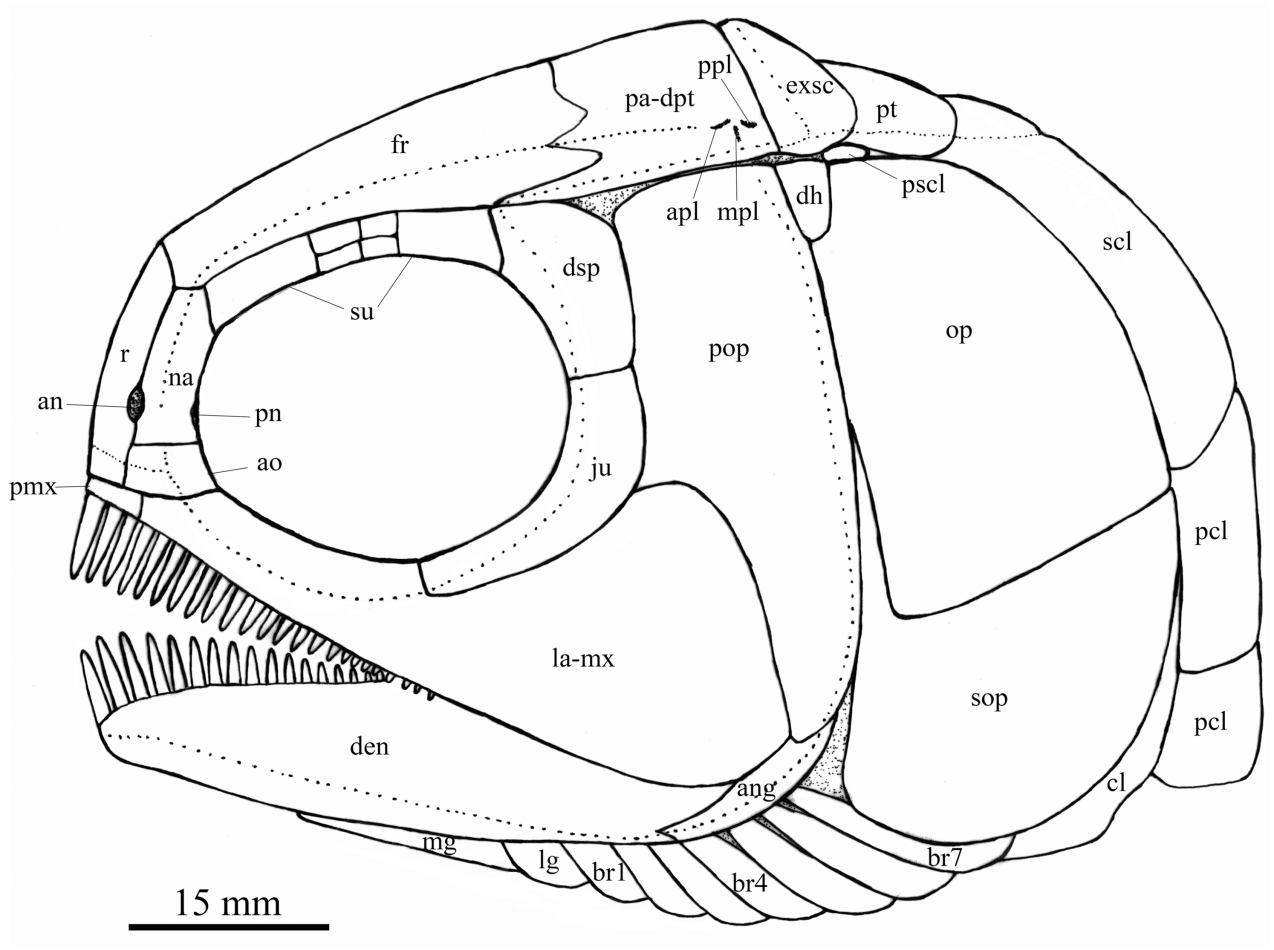
Reconstruction of skull and pectoral girdle. Reconstruction of skull and pectoral girdle of *Feroxichthys yunnanensis* gen. et sp. nov.

**Figure 5:**
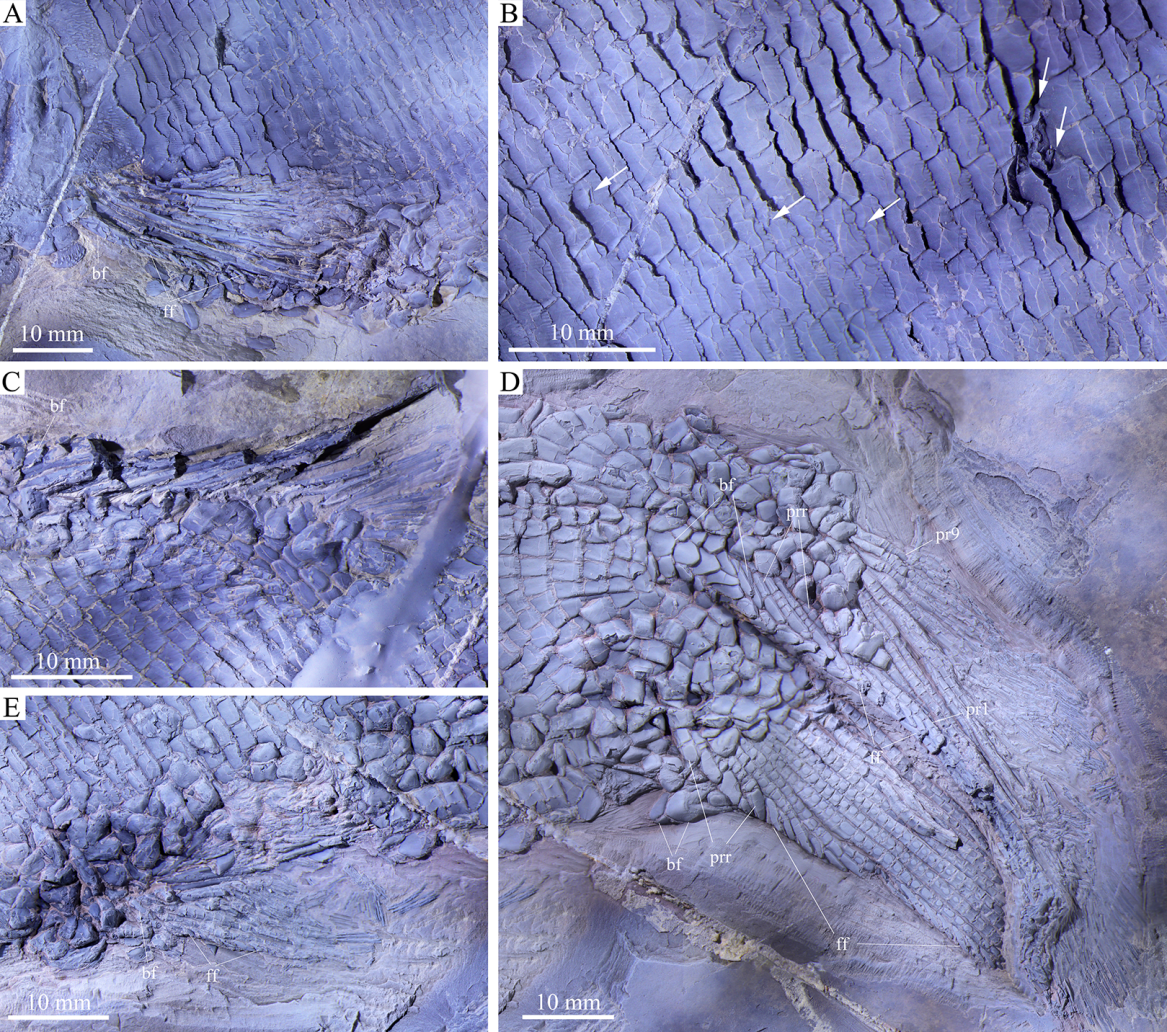
Fins and scales. Fins and scales of *Feroxichthys yunnanensis* gen. et sp. nov., V25692 (holotype). (A) Left pectoral fin, (B) scales in the anterior flank region, with arrows indicating short slits in lateral line scales and peg-socket articulations between scales, (C) dorsal fin, (D) caudal fin and (E) anal fin.

**Etymology.** The specific epithet is derived from Yunnan Province, where the specimen was collected.

**Holotype.** IVPP V 25692, a nearly complete specimen with part of dorsal and pelvic fins and a small region of flank scales missing.

**Locality and horizon:** Luoping, Yunnan, China; Second (Upper) Member of Guanling Formation, Pelsonian (~244 Ma), Anisian, Middle Triassic (Zhang et al., 2009).

**Diagnosis:** A new colobodontid distinguished from other members of this family by the following features (autapomorphies, those unique among colobodontids, identified with an asterisk): rostral depth 62% of frontal length; absence of postrostral; fusion of parietal with dermopterotic (*); strong posterior process on parieto-dermopterotic (*); presence of six supraorbitals; fusion of lacrimal with maxilla (*); opercle 1.5 times deeper than subopercle; fusion of paired premaxillae with five large and strong, peg-like teeth (*); large peg-like teeth only on anterior portions of maxilla and dentary; seven pairs of branchiostegal rays (*); 14 dorsal fin rays; 13 anal fin rays; six basal fulcra and four segmented procurrent rays in dorsal lobe of caudal fin; scales relatively smooth without tubercles; and pterygial formula of D52/P29, A48, C74/T~80 (*).

### Description

**General morphology and size.** *Feroxichthys* gen. nov. has a blunt snout, a fusiform body and an abbreviated heterocercal caudal fin with the dorsal fin inserting slightly posterior to the origins of the pelvic fins. The holotype (Fig. 1), a single specimen, has a standard length (the length from the tip of the snout to the posterior extremity of the caudal peduncle) of 290 mm, and a total length of about 340 mm. The skull, strongly ornamented with dense ganoid tubercles and some striae, has a length of 82 mm. The greatest body depth (86 mm) lies midway between the posterior margin of the opercle and the origin of the dorsal fin. The postcranium cannot be completely reconstructed because the caudal fin is not well preserved and the dorsal and pelvic fins are partly missing. Additionally, the vertebral column and dorsal and anal pterygiophores are not visible due to the squamation in situ.

**Snout.** The canal-bearing bones in the snout region consist of a median rostral and paired nasals and antorbitals (Fig. 2). The rostral is large and shield-like, having a concave ventral margin, a rounded dorsal margin and slightly curved lateral margins. A small ventral (anterior) portion of the lateral margin is concave, suturing with the slightly convex medial margin of the antorbital. The remaining lateral margin of the rostral sutures with the medial margin of the nasal, and both margins are notched for the anterior nostril. The depth of the rostral is 62% of the length of the frontal. The anterior ethmoid commissure of the lateral line system is enclosed in the rostral (Figs. 2 and 3D), indicated by a dorsally convex line of small pores at the anteroventral portion of this bone.

The nasals are trapezoidal, contacting the antorbital ventrally, the frontal dorsally, and the anterior supraorbital posterolaterally. The ventral portion of its lateral margin forms part of the anterior orbital margin, bearing a notch indicating the position of the posterior nostril. The supraorbital sensory canal extends into the nasal from the frontal, and ends at the level of the nostril notches (Fig. 2).

The antorbitals are elongated and pentagonal, and partly defines the anteroventral margin of the orbit. The conjunction of the ethmoid commissure and the infraorbital canal is located near the center of this bone (Figs. 2, 3A and 3D).

**Skull roof.** The skull roofing bones include a pair of frontals, parieto-dermopterotics, and extrascapulars (Fig. 2). The medial suture between frontals is slightly curved, and the left frontal is larger than the right one in dorsal view. The most anteromedial part of the frontal has a narrow area overlapped by the posterior part of the rostral. Each parietal is fused with the dermopterotic. The suture between the parieto-dermopterotic and the frontal is zigzag-like. The right parieto-dermopterotic is slightly larger than the left one and the medial suture between them is curved.

Notably, the right parieto-dermopterotic bears a large posterior process, which tapers posteriorly with a straight lateral margin and a serrated median margin (Fig. 2). The length of this posterior process is slightly longer than the lateral margin of the extrascapular. The posterior process of the left parieto-dermopterotic is not exposed because of the overlap of the lateral portions of the extrascapular and posttemporal.

The supraorbital canal extends longitudinally through the frontal, runs into the parieto-dermopterotic and ends at the middle-posterior portion of this compound ossification (Figs. 2 and 3E). Three short pit-lines are present in the posterolateral area of the parieto-dermopterotic, including a slight curved anterior pit-line, a laterally extended middle one and a posterolaterlly extended posterior one. The temporal sensory canal runs longitudinally through the parieto-dermopterotic, indicated by a series of small pores parallel to the lateral margin of this bone. In addition, there are four relatively large pores near the base of the posterior process of the parieto-dermopterotic (Fig. 2).

A single pair of extrascapulars is present. They are roughly trapezoidal, as wide as the parieto-dermopterotic, with the supratemporal commissure running transversely through both extrascapulars (Figs. 2 and 3C).

**Circumorbital bones.** There are six rectangular or trapezoidal supraorbitals flanking the lateral margin of the frontal (Figs. 2 and 3); among them, the middle four are arranged into two horizontal lines. The first (anteriormost) supraorbital is the longest that is slightly larger than the last one, and each of the middle four elements nearly equals to one-sixth of the first in size.

The lacrimal has been fused with the infraorbital ramus of the maxilla. The lacrimo-maxilla encloses an anterior portion of the infraorbital sensory canal and forms the anteroventral margin of the orbit (Figs. 2 and 3). A small anterodorsal zone of the lacrimo-maxilla is overlapped by the antorbital.

The jugal defines the posteroventral margin of the orbit (Fig. 2). It is curved, slightly more expanded posterodorsally than anteroventrally, and contacts the dermosphenotic dorsally, the preopercle posteriorly, and the lacrimo-maxilla ventroposteriorly. The infraorbital sensory canal extends through the jugal parallel to the orbital margin of this bone and enters the dermosphenotic dorsally.

The dermosphenotics on both sides of the skull are discernable in lateral view: the right dermosphenotic is detached and fully exposed, and the left one in situ (Figs. 2 and 3). Each dermosphenotic is slightly shorter than the jugal, with a concave anteroventral margin and a rounded posterodorsal margin. The dermosphenotic has a relatively large anteroventral area overlapped by the last supraorbital and a small ventral area overlapped by the dorsal portion of the jugal. The sensory canal extends from the anterodorsal corner of the dermopterotic into the anterior tip of the parieto-dermopterotic.

Two sclerotic bones are partly preserved near the dorsal rim of the orbit; both are thin and slightly curved (Fig. 2).

**Jaws.** The paired premaxillae are fused into a median ossification, bearing a short triangular nasal process on each side (Figs. 2 and 3). A foramen for the olfactory nerve is absent in the nasal process, as in other stem-neopterygians. Four large teeth and a tooth socket indicate that the fused premaxillae had five teeth along its oral margin. They are long and peg-like with an acuminate acrodine apex, and the median one is the longest and strongest.

The maxilla has an elongated infraorbital ramus fused with the lacrimal (described above) and a sub-triangular postorbital blade that is slightly deeper than the orbit (Figs. 2 and 3). The length of the maxilla is 2.5 times its maximum depth. The posterior margin of the maxilla is rounded, and the tooth-bearing margin nearly straight. Eighteen peg-like teeth are present only in the anterior half of the maxilla. They gradually reduce in length posteriorly; the first, longest tooth is nearly as long as the lateral one in the premaxilla.

The lower jaw is wedge-shaped with two elements, dentary and angular, discernable in lateral view (Fig. 2). The supra-angular, commonly present in other stem-neopterygians, is not exposed. The teeth in the dentary are peg-like, similar to those of the maxilla in length. The anterior three teeth are nearly equal in size and slightly inclined anteriorly, and others gradually reduce in length posteriorly. The angular is small and elongated, accounting 1/4 the length of the lower jaw. The angular and dentary carry the mandibular canal forward from the preopercle. The sutures between coronoids and prearticular on the medial surface of the lower jaw cannot be identified through the X-ray scanning, but molariform teeth on these bone are discernable; they are blunt or rounded, and those in the posteromedial region of the prearticular are the largest (Fig. 3E).

**Parasphenoid and palatoquadrate.** The parasphenoid and palatoquadrate are partly discernable through the orbit (Fig. 2). The exposed portion of the parasphenoid is elongated and that of the palatoquadrate is sub-circular. As showed by the X-ray scanning (Fig. 3F), dense rounded molariform teeth are present on the oral margins of pterygoid bones.

**Opercular series and dermohyal.** The preopercle is club-shaped and vertically oriented, tapering ventrally (Fig. 2). It has a rectangular dorsal part and a roughly trapezoidal ventral part with a small triangular anterior process inserting between the jugal and maxilla. The preopercular sensory canal is indicated by a vertical line of small pores close to the posterior margin of this bone. A small and nearly trapezoidal dermohyal is wedged between the preopercle and the opercle.

The opercle is large and nearly trapezoidal, with a depth/length ratio of 1.6. It has nearly straight anterior and ventral margins and convex dorsal and posterior margins. The subopercle is sickle-shaped, bearing a deep anterodorsal process (Figs. 2 and 3). This process is 44% of the depth of the opercle. Excluding this process, the subopercle is 66% of the depth of the latter bone. An interopercle is absent, as in other stem-neopterygians.

**Branchiostegal rays and gulars.** A complete series of seven left branchiostegal rays and several right ones are discernable (Fig. 2). They are moderately elongated and sub-triangular, tapering anteriorly. The first (anteriormost) branchiostegal ray is the narrowest, half of the width of the second one; the remaining rays are nearly as wide as the second.

A pair of lateral gulars is well exposed; each is elongated and plate-like, twice as wide as the first branchiostegal ray, bearing a pit-line in its anterolateral region (Fig. 2). The median gular remains unknown because of incomplete preservation; more specimens are needed to determine if it is present as commonly in other stem-neopterygians (Fig. 4).

**Paired girdles and fins.** A posttemporal, a presupracleithrum, a supracleithrum, a cleithrum and two postcleithra are discernable on each side in the pectoral girdle. The posttemporal is trapezoidal, nearly half as wide as the extrascapular. The lateral line pierces the anterolateral portion of the posttemporal and extends posteroventrally into the dorsal portion of the supracleithrum. The presupracleithrum is small and sub-circular, contacting the opercle ventrally and the posttemporal and extrascapular dorsally (Fig. 2).

The supracleithrum is plate-like, nearly as deep as the opercle. Most of the cleithrum is overlapped by the opercle, subopercle and branchiostegal rays and its complete shape remains unknown. There are two postcleithra associated with the cleithrum; the dorsal one is rhomboid, and the ventral is trapezoidal, having nearly half the depth of the dorsal.

The pectoral fins insert low on the body, and each is composed of about ten distally segmented rays. The first ray is unbranched, preceded by a basal fulcrum and a series of small, leaf-like fringing fulcra. The remaining rays are branched distally. The basal fulcrum is ornamented with elongated tubercles but the rays are smooth on their surfaces (Fig. 5A).

The pelvic girdles are not exposed. The pelvic fins insert at the 29^th^ vertical scale row, and each is composed of five distally segmented and branched rays, preceded by two basal fulcra and a series of fringing fulcra.

**Median fins.** The dorsal fin originates above the 52^th^ vertical scale row. It is composed of 14 rays, preceded by a basal fulcrum (Fig. 5C). Among them, the anterior three rays are incompletely preserved with their distal portions missing, and the posterior rays are distally segmented and branched. Because of incomplete preservation, fringing fulcra are unknown in the dorsal fin. More specimens are needed to determine if they are present as in other colobodontids.

The anal fin originates below the 48^th^ vertical scale row, composed of 13 distally segmented rays. The first ray is unbranched, preceded by a basal fulcrum and a series of fringing fulcra, and the remaining rays are branched distally. The rays are smooth on the surface (Fig. 5E).

The abbreviated heterocercal caudal fin is not fully exposed. The dorsal lobe flips downwards and partly covers the ventral one, and thus, the total number of rays cannot be counted. The dorsal lobe includes four segmented procurrent rays and at least nine principal rays (Fig. 5D). In addition, there are six epaxial basal fulcra in the dorsal lobe; the anterior three are unpaired, the rest paired. Two hypaxial basal fulcra (a median anterior and paired posterior ones; preserved back to front) and four segmented procurrent rays are present in the ventral lobe. Two leaf-like fringing fulcra are discernable between the last hypaxial procurrent ray and the last principal ray, and also between the last principal ray and the last branched ray. Most of other hypaxial fringing fulcra are associated to the ventral margin of the penultimate branched ray in the ventral lobe. However, the fringing fulcra in the dorsal lobe are incompletely preserved with only anterior four paired ones discernable. The surfaces of caudal fin rays are ornamented with rounded or elongated tubercles.

**Scales.** The scales are rhomboid and ganoid with serrated posterior margins (Figs. 1 and 5). They are arranged in about 80 vertical rows along the lateral line. In the 20^th^ vertical row of scales, 16 and 14 scales are present above and below the lateral line on each side of the body, respectively. The scales in the anteroventral flank region are the largest, nearly twice as deep as long, and they gradually become shorter dorsally, ventrally and posteriorly. The scales are relatively smooth on the surface except for some parallel ridges extending from the serrations in their posterior margin. A short slit is present in some lateral line scales (Fig. 5B), which probably represents the opening of the pit organ that is separate and independent from the lateral line canal (Schultze, 1966). As common for other early actinopterygians, a peg-socket articulation is discernable from several scales in the anterior flank region (Fig. 5B).

## Discussion

### Phylogenetic affinities

**Figure 6:**
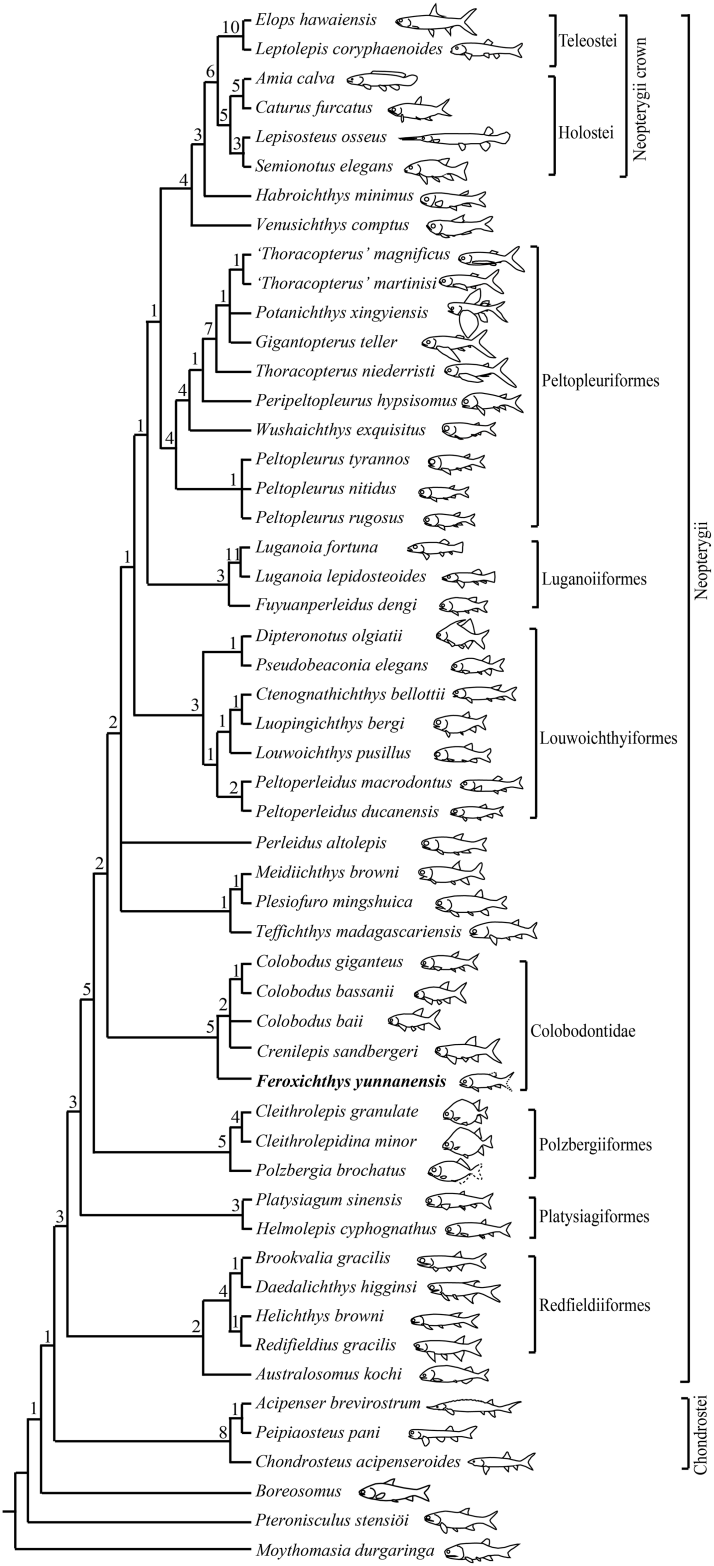
Strict consensus of 24 most parsimonious trees. Strict consensus of 24 most parsimonious trees (tree length = 341 steps, consistency index = 0.4663, retention index = 0.7708), illustrating the phylogenetic position of *Feroxichthys yunnanensis* gen. et sp. nov. within the Neopterygii. Numbers above nodes indicate Bremer decay indices. For character descriptions and data matrix, see the Supplemental Material.

My analysis resulted in 24 most parsimonious trees (tree length = 341 steps, consistency index = 0.4663, retention index = 0.7708), a strict consensus of which is presented in Fig. 6. In this cladogram, *Feroxichthys* gen. nov. is recovered at the base of the Colobodontidae (new usage here); the Colobodontidae is nested above the Polzbergiiformes on the Neopterygii stem and consists of the sister group of an unresolved polytomy involving *Teffichthys-*like taxa, *Perleidus altolepis* and the clade Lowoichthyiiformes plus more derived neopterygians.

The Colobodontidae is evidently a stem lineage of neopterygian fishes, possessing several derived features of this clade above the platysiagiform level, such as a vertical suspensorium, three or more supraorbitals (reduced or secondarily lost in some derived forms), a more ventrally extended dermosphenotic than the dermopterotic (reversal in halecomorphs), and presence of segmented procurrent rays in the dorsal lobe of the caudal fin (independently evolved in the Pholidopleuriformes–Redfieldiiformes clade, secondarily lost in *Teffichthys*-like taxa and most crown neopterygians). It is more derived than the Polzbergiiformes in having dorsal and anal fin rays that are segmented only at the distal region, and a 1:1 ratio of fin rays to endoskeletal radials in dorsal and anal fins (unknown in *Feroxichthys* gen. nov. because of preservation). However, the Colobodontidae lacks the derived features of *Teffichthys-*like taxa, *Perleidus* and more derived neopterygians: absence of the dermosphenotic/preopercle contact, presence of the opercle no larger than the subopercle (reversal in Peltopleuriformes and more derived neopterygians), no more than six pairs of branchiostegal rays (reversal in some crown neopterygians), and no more than 24 principal rays in the caudal fin (reversal in *Fuyuanperleidus* and derived louwoichthyids). It further lacks synapomorphies of crown neopterygians, such as presence of a reduced rostral, a supramaxilla, an interopercle, and a mobile maxilla free from the preopercle (Patterson, 1973, 1982).

The monophyly of the Colobodontidae is supported by five synapomorphies: anterior portions of frontals partly separated by broad rostral or postrostral bone (independently evolved in basal actinopterygians and living chondrosteans); presence of a deep anterodorsal process of the subopercle (independently evolved in holosteans); presence of multiple supraorbitals arranged in more than one horizontal rows (independently evolved in and *Caturus*; reversal in *Colobodus bassanii*), absence of the suborbital (independently evolved in platysiagiforms, *Pseudobeaconia*, *Habroichthys* and some crown neopterygians), and principal rays of caudal fin ornamented with rounded ganoid tubercles (uniquely derived among early neopterygians). *Feroxichthys* gen. nov. is recovered as a basal member of the Colobodontidae because it possesses above synapomorphies but lacks two derived features of other members (*Colobodus* and *Crenilepis*) of this family: presence of a postrostral (independently evolved in *Pseudobeaconia*) and strong ornamentation of tubercles and longitudinal ridges of ganoine on scales (independently evolved in some basal actinopterygians). Above *Feroxichthys* gen. nov., *Colobodus bassanii* is recovered sister to *C. giganteus*, supported by presence of two or three segmented procurrent rays in the dorsal lobe of the caudal fin. However, their relationships with *C. baii* and *Crenilepis* are not resolved and need further studies.

Consistent with previous hypotheses (Xu, Gao & Coates, 2015; Xu, Ma & Zhao, 2018; Wen et al., 2019; Xu, 2020a), the results suggest that the ‘Perleidiformes’ or even the ‘Perleididae’ are paraphyletic groups composed of a series of independent stem neopterygian lineages. Notably, the fuyuanperleidid ‘perleidiform’ *Fuyuanperleidus* is recovered sister to the luganoiid *Luganoia* (represented by *Luganoia lepidosteoides* and *L. fortuna*), supported by the presence a fused parieto-dermopterotic (independently evolved in *Feroxichthys* gen. nov. and thoracopterids), a fused lacrimo-maxilla (independently evolved in *Feroxichthys* gen. nov.), absence of fringing fulcra (independently evolved in thoracopterids, habroichthyids and most crown neopterygians), and greatly deepened anterior flank scales corresponding to two or three horizontal rows of relatively shorter scales posteriorly. Thus, the Fuyuanperleididae is referred to the Luganoiiformes here. Additionally, the ‘perleidid’ *Peltoperleidus* (represented by *Peltoperleidus ducanensis* and *P. macrodontus*) is recovered at the base of the Louwoichthyidae (Louwoichthyiformes). However, the interrelationships between three species of *Peltopleurus* are not resolved. The phylogenetic relationships concerning other taxa are similar to those proposed by Xu (2020a) and are unnecessary to repeat here.

### Character comparisons

Besides the features listed above, *Feroxichthys* gen. nov. is easily distinguished from *Colobodus* and *Crenilepis* within the Colobodontidae, or more generally, from other stem neopterygians in the following aspects:

(1) Presence of fused premaxillae. *Feroxichthys* gen. nov. possesses a median, fused premaxillae with five long peg-like teeth on the oral margin of this ossification. Similar conditions are present in the East Greenland ‘*Perleidus*’ (Patterson, 1975) and some thocopterids (Tintori & Sassi, 1992; Xu et al., 2012). In luganoiids, the premaxillae are further fused with the rostral (Bürgin, 1992). By contrast, other stem neopterygians (including other colobodontids) generally have a pair of independent premaxillae. Patterson (1975) argued that the premaxillae (with two teeth on each side) of the East Greenland ‘*Perleidus*’ were fused in late ontogeny. However, the fused premaxillae of *Feroxichthys* gen. nov. has an odd number (five) of teeth with the median one being the largest, indicating that the fusion in this taxon was probably developed very early in ontogeny.

(2) Fusion of dermopterotic with parietal. *Feroxichthys* gen. nov. has a pair of parieto-dermopterotics, differing from the conditions in other colobodontids but resembling those in several other stem-neopterygians, for example the perleidid *Endennia* (Lombardo & Brambillasca, 2005), thoracopterids (Griffith, 1977; Tintori & Sassi, 1992; Xu et al., 2012; Xu, Zhao & Shen, 2015) and luganoiids (Brough, 1939; Bürgin, 1992). Outside of neopterygians, this fusion is also present in the living *Polypterus* (Allis, 1909). A further fusion of frontals, parietals and dermopterotics into a single broad skull roofing plate is discernable in some small-sized stem neopterygians (e.g. habroichthyids and peltopleurids) and basal teleosts (Arratia, 2013).

(3) Posterior process of parieto-dermopterotic. The parieto-dermopterotic of *Feroxichthys* gen. nov bears a triangular posterior process, a uniquely derived feature among stem neopterygians. This process is absent in other well-studied stem neopterygians with parieto-dermopterotics (e.g. thoracopterids and luganoiids). A similar process is otherwise present in the parieto-dermopterotic of the living *Polypterus*, and it is related to the insertion of trunk muscles (Allis, 1909). Analogously, the process of the parieto-dermopterotic in *Feroxichthys* gen. nov. may have had the same function.

(4) Fusion of lacrimal with maxilla. *Feroxichthys* gen. nov. is peculiar in having a fused lacrimo-maxilla. This condition is unknown in other colobodontids, and consequently represents an autapomorphy of the genus within this family. A similar condition is otherwise known only in luganoiids (Brough, 1939; Bürgin, 1992; Xu, 2020b) and fuyuanperleidids within the Neopterygii. However, *Feroxichthys* gen. nov. slightly differs from luganoiids in that the lacrimo-maxilla does not contribute to the composition of the anterior margin of the orbit. Outside of the Neopterygii, an infraorbital/maxilla fusion is present in the living *Polypterus*, in which the sixth and seventh infraorbitals are fused with the maxilla (Rizzato et al., 2020).

(5) Opercular series. The opercle of *Feroxichthys* gen. nov. is 1.5 times as large as its subopercle, similar to the conditions in other colobodontids (Mutter, 2002; Cartanyà et al., 2015), fuyuanperleidids (Geng et al., 2012; Sun et al., 2012) and peltopleurids (Bürgin, 1992; Xu, Ma & Zhao, 2018). An even larger opercle is present in thoracopterids, venusichthyids and habroichthyids (Griffith, 1977; Bürgin, 1992; Lin et al., 2011; Xu et al., 2012; Xu & Zhao, 2016). By contrast, the opercle is nearly equal to or smaller than the subopercle in *Perleidus*, *Teffichthys-*like taxa (e.g. *Teffichthys* and *Plesiofuro*), pseudobeaconiids, louwoichthyids and luganoiids (Lehman, 1952; Hutchinson, 1973a, 1973b; Bürgin, 1992; Lombardo, 2001; López-Arbarello & Zavattieri, 2008; Xu, Zhao & Shen, 2015; Marramà et al., 2017; Xu, 2020a, 2020b). Additionally, the subopercle of *Feroxichthys* gen. nov. bears a deep anterodorsal process (44% of the depth of the opercle), resembling the conditions in other colobodontids (Mutter, 2002; Cartanyà et al., 2015) and many holosteans (Grande & Bemis, 1998; Xu, 2019). This process, if present, is rudimentary or short in other stem neopterygians and teleosts.

(6) Gulars and branchiostegal rays. Unambiguous lateral gulars are present in *Feroxichthys* gen. nov. because these bones are well distinguishable from the adjacent branchiostegal ray by their shape and size, and perhaps most importantly, the associated pit-line in their anterolateral portion. Similar pit-lines were previously known in the lateral gulars of some *Teffichthys*-like taxa (Lehman, 1952; Xu, Zhao & Shen, 2015). A pair of possible lateral gulars was reconstructed in other colobodontids (Mutter, 2002) according to their sizes larger than the adjacent branchiostegal rays. It remains unknown if the median gular is present in *Feroxichthys* gen. nov. because of incomplete preservation. As the median gular is commonly present in other stem-neopterygians, this bone was tentatively reconstructed here (Fig. 4). As for branchiostegal rays, the number in *Feroxichthys* gen. nov. (seven pairs, another autapomoprhy of this taxon among colobodontids) is significantly less than that in other colobodontids (13–18 pairs; Mutter, 2002, 2004). Even less branchiostegal rays are present in *Perleidus* (five or six pairs), pseudobeaconiids, venusichthyids and louwoichthyids (two or three pairs), and habroichthyids (single pair).

(7) Caudal fin. *Feroxichthys* gen. nov. has four segmented procurrent rays in the dorsal lobe of the caudal fin. This number is less than that in *Crenilepis* and *Colobodus baii* (about eight) but slightly larger than that in *C. giganteus* and *C. bassanii* (two or three). Four or more procurrent rays are otherwise present in pholidopleuriforms, many redfieldiiforms, *Perleidus*, derived louwoichthyids, luganoiiforms and most peltopleuriforms but they are lost in platysiagiforms, several redfieldiiforms, *Peltoperleidus* and *Teffichthys*-like taxa. Additionally, fringing fulcra are present in both lobes of the caudal fin of *Feroxichthys* gen. nov., differing from those of thoracopterids, fuyuanperleidids, luganoiids and habroichthyids, in which the fringing fulcra are lost (Griffith, 1977; Bürgin, 1992; Lin et al., 2011; Geng et al., 2012; Sun et al., 2012; Xu, Gao & Coates, 2015; Xu, 2020b).

### Implications

The discovery of *Feroxichthys* gen. nov. extends the geological range of Chinese colobodontids from the early Middle Triassic (Anisian) of Panxian (*Colobodus baii*) and the late Middle Triassic (Ladinian) of Xingyi (*C. wushaensis*) in Guizhou Province into the early Middle Triassic (Anisian) of Luoping in Yunnan Province. A detailed geological survey indicates that the fossiliferous level of *Feroxichthys* gen. nov. (middle part the Second Member of the Guanling Formation) is slightly lower than that of *C. baii* (upper part of the Second Member of the Guanling Formation), although both are located at the same stage (Pelsonian, Anisian) of the Middle Triassic by the conodont analyses (Sun et al., 2006, 2016; Zhang et al., 2009). Early colobodontids in Europe are recovered near the Anisian/Ladinian boundary of the Besano Formation exposed in the Monte San Giorgio area (Bürgin, 1996; Mutter, 2002), and thus, are slightly younger than their relatives (*Feroxichthys* gen. nov. and *C. baii*) from South China. Currently, *Feroxichthys* gen. nov. documents the oldest record of colobodontids on earth.

In feeding apparatus, *Feroxichthys* gen. nov. is structurally similar to other colobodontids (Bürgin, 1996; Mutter, 2002, 2004): a nearly vertically oriented suspensorium, jaws composed of small premaxillae, club-shaped maxillae and wedge-shaped lower jaws with straight tooth-bearing margins, and dentition including sharp peg-like teeth on the premaxilla, maxilla and dentary, and blunt molariform teeth on the coronoids, prearticular and pterygoids. Such dentition combining grasping and crushing morphologies is common in living durophagous fishes (Helfman et al., 2009), which usually employ anterior peg-like or conical teeth for initial prey capture before food was passed posteriorly to flatted or rounded molariform teeth for some sort of crushing in the oral cavity. Analogously, a durophagous diet is suggested for *Feroxichthys* gen. nov., as previously suggested for other colobodontids and several ‘perleidids’ (Bürgin, 1996; Mutter, 2002, 2004). A notable difference is that the anterior peg-like teeth of *Feroxichthys* gen. nov. are much longer and stronger than those of other colobodontids, enabling a more powerful prey capture than the latter taxa.

The Luoping Biota in the Middle Triassic Yangtze Sea included a rich variety of potential invertebrate prey of *Feroxichthys* gen. nov., such as crustaceans, gastropods, brachiopods, bivalves, ammonoids and millipedes (Hu et al., 2011; Feldmann et al., 2012; Huang et al., 2013). Additionally, given its large size, *Feroxichthys* may have also preyed upon many other heavily armoured but small-sized neopterygian fishes (e.g, peltopleurids, habroichthyids, venusichthyids and platysiagids). These invertebrates and small-sized fishes are mainly primary consumers in the food web of the Luoping Biota (Hu et al., 2011; Benton et al., 2013; Xu & Ma, 2016; Xu & Zhao, 2016; Wen et al., 2019).

The discovery of *Feroxichthys* gen. nov. adds new information on the trophic structure of this biota. Along with *Feroxichthys* gen. nov., large-sized predators of ray-finned fishes from the Anisian Luoping Biota are known by saurichthyids (Wu et al., 2011) and some holosteans (e.g. *Robustichthys*; Xu, Zhao & Coates, 2014; Xu, 2019). They are hypothesized to be the secondary consumers, forming the middle part of the food web of this biota. *Feroxichthys* gen. nov. represents the first evidence of large durophagous predatory fishes from this biota. Even larger predatory fishes, e.g. birgerids (up to 2 m in body length) recovered from many other marine Triassic deposits (Stensiö, 1932; Nielsen, 1949; Romano et al., 2017; Ni et al., 2019), remain unknown from Luoping. Similar to those from the Middle Triassic communities in Europe, the top predators (tertiary consumers) from the Luoping Biota are large marine reptiles (e.g. nothosaurs, ichthyosaurs and protorosaurs), and some of them are gigantic apex predators (e.g. *Nothosaurus zhangi*; Liu et al., 2014), which have a body length of about 5–7 m and could prey on large fishes (probably including *Feroxichthys* gen. nov.) or even smaller marine reptiles from the same biota (Hu et al., 2011; Benton et al., 2013; Liu et al., 2014). These marine reptiles and neopterygian fishes (stem taxa and holosteans) are new clades in the aftermath of the end-Permian mass extinction. Their occurrences added new trophic levels, indicating that a healthy trophic structure from primary producer to top predator had already been recovered in early Middle Triassic marine ecosystems (Chen & Benton, 2012).

## Conclusion

The discovery of *Feroxichthys* gen. nov. documents the first evidence of large-sized stem neopterygians from the Anisian Luoping Biota, providing new insights into the morphological diversity, body size and phylogeny of early neopterygians. Phylogenetic analysis resolves it as a basal colobodontid, which possesses diagnostic features of this clade but is easily distinguished from other colobodontids with some derived features (e.g. fusion of paired premaxillae, fusion of the lacrimal with the maxilla and a fused parieto-dermopterotic with a strong posterior process). *Feroxichthys* gen. nov. represents the oldest colobodontid on earth, suggesting that the origin of this clade occurred no later than the Pelsonian, Anisian (~244 Ma). As a large durophagous predator previously unknown from Luoping, the new finding adds our understanding on the complex trophic structure of this biota, which renders support that a stable, complex ecosystem has re-emerged in the early Middle Triassic, ~8 Myr after the end-Permian period mass extinction.

## Supplemental Information

10.7717/peerj.10229/supp-1 Supplemental Information 1 Taxa and principal sources of data, characters and data matrix.

10.7717/peerj.10229/supp-2 Supplemental Information 2 Data matrix.

## Anatomical Abbreviations

adp: Anterodorsal process of subopercle
an: Anterior nostril
ang: Angular bone
ao: Antorbital bone
apl: Anterior pit-line
br: Branchiostegal rays
cl: Cleithrum
den: Dentary
dh: Dermohyal
dsp: Dermosphenotic
etc: Ethmoid commissure
exsc: Extrascapular bone
fr: Frontal bone
ioc: Infraorbital canal
la-mx: Lacrimo-maxilla
lg: Lateral gular
ju: Jugal
mg: Median gular bone
n: Nasal bone
mpl: Middle pit-line
npp: Nasal process of premaxilla
ppt: Posterior process of parieto-dermopterotic
op: Opercle
pa-dpt: Parieto-dermopterotic
pas: Parasphenoid
pcl: Postcleithrum
ppl: Posterior pit-line
ppr: Procurrent ray
pmx: Premaxilla
pn: Posterior nostril
pscl: Presupracleithrum
pop: Preopercle
pq: Palatoquadrate
pt: Posttemporal
r: Rostral bone
sc: Scale
scl: Supracleithrum
stc: Supratemoral commissure
scr: Sclerotic ring
soc: Supraorbital canal
sop: Subopercle
su: Supraorbital bone

## Additional Information and Declarations

### Data Availability

The following information was supplied regarding data availability: The raw data are available in the Supplemental Files. The specimen (IVPP V 25692) is curated at the fossil collections of the Institute of Vertebrate Paleontology and Paleoanthropology (IVPP), Chinese Academy of Sciences in Beijing, China.

### New Species Registration

The following information was supplied regarding the registration of a newly described species: Publication LSID: urn:lsid:zoobank.org:pub:868619C7-6001-4BCE-A2B9-6C3CC7192CA5. *Feroxichthys* gen. nov. LSID: urn:lsid:zoobank.org:act:3E8B9492-862D-458D-9EF8-9C68F6C340B1. *Feroxichthys yunnanensis* gen. et sp. nov. LSID: urn:lsid:zoobank.org:act:F4ED5C97-1601-41D8-A520-EF2CBB4AF4F5.
