# Supplementary material for: Feroxichthys yunnanensis gen. et sp. nov. (Colobodontidae, Neopterygii), a large durophagous predator from the Middle Triassic (Anisian) Luoping Biota, eastern Yunnan, China
Source: PeerJ. 2020 Oct 20;8:e10229. doi: 10.7717/peerj.10229 (PMC7583626; doi:10.7717/peerj.10229)
Supplement: Supplemental Information 1 [file peerj-08-10229-s001.pdf]

Online Supplementary Material

Feroxichthys yunnanensis gen. et sp. nov. (Colobodontidae, Neopterygii), a large durophagous predator from the Middle Triassic (Anisian) Luoping Biota, eastern Yunnan, China

Table of Contents

1. Taxa and principal sources of data
2. Supplementary figures
3. Character list
4. Data matrix
5. References to supplementary information

1. Taxa and principal sources of data

FMNH, Field Museum of Natural History, Chicago, USA

IVPP, Institute of Vertebrate Paleontology and Paleoanthropology, Chinese Academy of Sciences, Beijing, China

NHMUK, Natural History Museum, London, UK;

PIMUZ, Paläontologisches Institut und Museum, Universität Zürich, Zürich, Switzerland

Moythomasia durgaringa: Gardiner, 1984

Australosomus kochi: Nielsen, 1949; NHMUK P17141–17143, 17156, 17157, 17160, 17161, 20940–20945

Acipenser brevirostrum: Hilton et al., 2011

Amia calva: Grande and Bemis, 1998

Boreosomus piveteaui: Nielsen, 1942

Brookvalia gracilis: Hutchinson, 1973b

Caturus furcatus: FMNH UC2057; Patterson, 1975; Grande and Bemis, 1998

Chondrosteus acipenseroides: Hilton and Forey, 2009

Cleithrolepidina minor: Hutchinson, 1973b

Cleithrolepis granulate: Wade, 1935; Hutchinson, 1973b

Colobodus baii: Sun et al., 2008; IVPP V19974

Colobodus bassanii: Mutter, 2002, 2004

Colobodus giganteus: Cartanyà et al., 2015

Crenilepis sandbergeri: Mutter, 2002, 2004

Ctenognathichthys bellottii: Bürgin, 1992

Daedalichthys higginsi: Hutchinson, 1973b

Dipteronotus olgiatii: Tintori, 1990

Elops hawaiensis: Forey, 1973

Fuyuanperleidus dengi: Geng et al., 2012; Sun et al., 2012

Gigantopterus teller: Griffith, 1977

Habroichthys minmius: Bürgin, 1992; PIMUZ T196, 233, 2917, 2883, 2884

Helichthys browni: Hutchinson, 1973b

Helmolepis cyphognathus: Neuman & Mutter, 2005

Lepisosteus osseus: Grande, 2010

Leptolepis coryphaenoides: Patterson, 1975; Arratia, 1999, 2013

Luganoia lepidosteoides: Bürgin, 1992

Luganoia fortuna: Xu, 2020b

Luopingichthys bergi: Sun et al., 2009

Louwoichthys pusillus: Xu, 2020a

Meidiichthys browni: Brough, 1931; Hutchinson, 1973b

Perleidus altolepis: Lombardo, 2001

Peipiaosteus pani: Zhou, 1992

Peltopleurus rugosus: Bürgin, 1992; PIMUZ T2904

Peltopleurus nitidus: Xu and Zhao, 2016

Peltopleurus tyrannos: Xu et al., 2018

Peltoperleidus ducanensis: Bürgin et al., 1991

Peltoperleidus macrodontus: Bürgin, 1992

Peripeltopleurus hypsisomus: Bürgin, 1992; PIMUZ T1211, 2150, 2869

Pseudobeaconia elegans: Hutchinson, 1973a; López-Arbarello and Zavattieri, 2008

Platysiagum sinensis: Wen et al., 2019

Plesiofuro mingshuica: Xu et al., 2015a

Polzbergia brochatus: Griffith, 1977

Potanichthys xingyiensis: Xu et al., 2012

Pteronisculus stensioi: Nielsen, 1942; NHMUK P16282, 16283, 16300, 163001, 16307–16308

Redifieldius gracilis: Schaeffer and McDonald, 1978

Semionotus elegans: Olsen and McCune, 1991; Cavin, 2010; Grande, 2010; López-Arbarello, 2012

Teffichthys (*'Perleidus'*) *madagascariensis*: NHMUK P16247, 16248, 19580–19584, 19587–19592, 19595–19599, 19603–19620, 19622, 19623; Lehman, 1952; Patterson, 1975; Marramà et al., 2017

Thoracopterus niederristi: Griffith, 1977; Lehman, 1979; NHMUK P1098

'Thoracopterus' magnificus: Tintori and Sassi, 1992

'Thoracopterus' martinisi: Tintori and Sassi, 1992

Venusichthys comptus: Xu and Zhao, 2016

Wushaichthys exquisitus: Xu et al., 2015b

Supplementary Figure

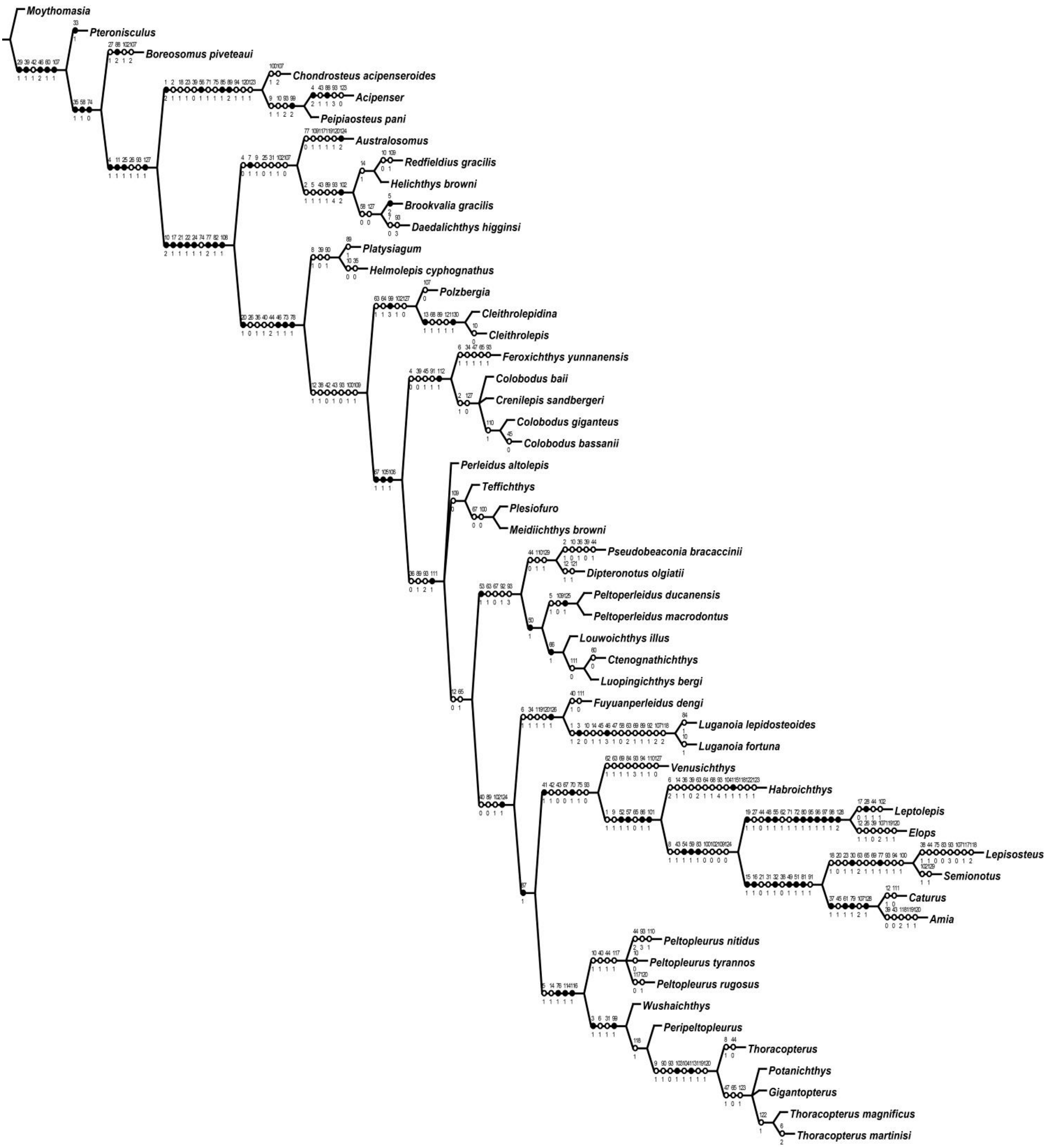

Supplementary Figure S1. Strict consensus of 24 MPTs (tree length = 341 steps, consistency index = 0.4663, retention index = 0.7708), illustrating phylogenetic position of *Feroxichthys yunnanensis* sp. nov. Character changes indicated with solid circles are unique.

2. Character list

The characters are mainly adopted or modified from previous publications on the phylogenetic relationships of the Neopterygii (A, Arratia, 2013; C, Coates, 1999; CA, Cloutier and Arratia, 2004; GS, Gardiner and Schaeffer, 1989; GML, Gardiner, Maisey and Littlewood, 1996; GSM, Gardiner, Schaeffer and Masserie, 2005); GB, Grande and Bemis, 1998; G, Grande, 2010; LZ, López-Arbarello and Zavattieri, 2008; L, López-Arbarello, 2012; P, Pinna, 1996; XG, Xu and Gao, 2011; XW, Xu and Wu, 2012; X, Xu et al., 2012; XGF, Xu, Gao and Finarelli, 2014; XZS, Xu, Zhao and Shen, 2015b; XGC, Xu, Gao and Coates, 2015a; XZ, Xu and Zhao, 2016; XM, Xu and Ma, 2016; XMZ, Xu, Ma and Zhao, 2018; XA, Xu, 2020a).

Skull Roof

(1) Rostral: large, shield-like or cap-like (0); small, much reduced or lost by fusion with other elements (1); irregularly shaped and anamestic (2). (Modified from GML19; XW17; XGC51; XM1; XMZ1; XA1)

(2) Postrostral: absent (0); present (1). (XA2)

(3) Frontal(s): elongate (0); laterally expanded (1); well constricted above orbit (2). (X33; XGC6; XM3; XMZ3; XA3)

(4) Contact relationships of frontals: anterior portions of frontals partly separated by median rostral bone (0); fully contact each other medially (1); completely separated by rostral bones (2).

(5) Supraorbital sensory canal: ending in parietal (0); ending in frontal (1); ending in dermopterotic (2). (Modified from X34; XGC7; XZS34; XM4; XMZ4; XA4)

(6) Distinct parietal: present (0); absent, fused with dermopterotic (1); absent, fused with frontal and dermopterotic (2). (Modified from XZS28; XM5; XMZ5; XA5)

Remarks: Based on personal observation of the holotype, the parietal is fused with the dermopterotic in *Fuyuanperleidus dengi* from the Middle Triassic (Anisian) of Luoping, Yunnan.

(7) Number of parietals: one pair (0); three or more (1). (Modified from XA6)

(8) Nasal bones: completely separated from each other by median rostral or rostral bones (0); joined or nearly joined in midline (1). (XM6; XMZ6; XA7)

(9) Nasal bone forming part of orbital margin: present (0); absent (1). (XZ55; XM7; XMZ7; XA8)

(10) Number of extrascapulars: two pairs or more (0); three (1); one pair (2). (Modified from X16; XGC8; XM23; XMZ23; XA9)

(11) Intertemporal: present (0); absent (1). (Modified from GSM17; XGC10; XM24; XMZ24; XA10)

(12) Ratio of dermopterotic length to parietal length: less than two (0); two or more (1).

(13) Accessory dermopterotic: absent (0); present (1). (XGC59; XM25; XMZ25; XA11)

(14) Dermopterotic/preopercle contact: present (0); absent (1). (XM26; XMZ26; XA12)

Neurocranium

(15) Sphenotic with small dermal component: absent (0); present (1). (G23; XW16; XM8; XMZ8; XA13)

(16) Pterotic: present (0); absent (1). (GML2; GMC47; XM9; XMZ9; XA14)

(17) Opisthotic: present (0); absent (1). (G33; XGC48; XM10; XMZ10; XA15)

(18) Intercalar: present (0); absent (1). (GML4; XM11; XMZ11; XA16)

(19) Supraoccipital: absent (0); present (1). (G28; XGC49; XM12; XMZ12; XA17)

(20) Post-temporal fossa: absent (0); present (1). (GS28; XGC1; XM13; XMZ13; XA18)

(21) Sub-temporal fossa: absent (0); present (1). (GS29; XGC2; XM24; XMZ14; XA19)

(22) Dilatator fossa: absent (0); present (1). (GS31; XGC3; XM15; XMZ15; XA20)

(23) Posterior myodome: present (0); absent (1). (Modified from GS10; GSM6; XM16; XMZ16; XA14; XA21)

(24) Anterodorsal myodome: present (0); absent (1). (XGF8; XZ8; XM17; XMZ17; XA14; XA22)

(25) Parasphenoid: short, terminates at otic fissure (0); long, extends across otic fissure (1). (Modified from GSM8; XGC4; XM18; XMZ18; XA23)

(26) Basipterygoid process: present (0); absent (1). (GSM12; XGC5; XM19; XMZ19; XA24)

(27) Internal carotid foramen on parasphenoid: absent (0); present (1). (GML14; XGC53; XM20; XMZ20; XA25)

(28) Efferent pseudobranchial foramen on parasphenoid: absent (0); present (1). (GML15; XGC54; XM21; XMZ21; XA26)

(29) Pineal foramen: present (0); absent (1). (XGF113; XA27)

Circumorbital Bones

(30) Anterior infraorbital bone(s): absent (0); present (1). (Modified from G21; XW24; XGC40; XM27; XMZ27; XA28)

(31) Antorbital (when it is not fused with premaxilla): small, shorter than nasal (0); enlarged, nearly equal to or deeper than nasal (1). (Modified from XA29)

(32) Tube-like canal bearing anterior arm of antorbital: absent (0); present (1). (G12; XW19; XGC43; XM28; XMZ28; XA30)

(33) Anterior part of lacrimal bearing teeth: absent (0); present (1). (Modified from XA31)

(34) Lacrimal: independent (0); fused with maxilla (1).

Remarks: Based on personal observation on the holotype, the lacrimal is fused with the maxilla in *Fuyuanperleidus dengi* from the Middle Triassic (Anisian) of Luoping, Yunnan.

(35) Dermosphenotic/nasal contact: present (0); absent (1). (Modified from GS19; XGC11; XM29; XMZ29; XA32)

Remarks: In *Helmolepis cyphognathus*, the triangular 'supraorbital' is better interpreted as a dermosphenotic because its shape and position (Neuman & Mutter, 2005).

(36) Dermosphenotic/preopercle contact: absent (0); present (1). (XA33)

(37) Dermosphenotic attachment to skull roof in adult-sized individuals: loosely attached on the skull roof or hinged to the side of skull roof (0); firmly sutured into skull roof, forming part of it (1). (GB56; XGC13; XM31; XMZ31; XA34)

(38) Position of dermosphenotic relative to dermopterotic (intertemporal plus supratemporal): located at nearly same horizontal level of dermopterotic (0); located below dermopterotic (1).

(39) Suborbital(s): absent (0); present (1). (Modified from GS9; XM35; XMZ35; XA35)

Remarks: The coding (1) for *Australosomus* is based on personal observation on NHMUK 17157.

(40) Positions of suborbital(s): extending below dermosphenotic (0); located posterior to dermosphenotic only (1). (Modified from XA36 and 37)

(41) Number of infraorbitals between antorbital and dermosphenotic: three or less (0); four or more (1). (Modified from GS21; XGC16; XM33; XMZ33; XA38)

(42) Postinfraorbital(s): absent (0); present (1). (XA39)

(43) Supraorbital: absent (0); present (1). (Modified from GS14; XGC17; XM34; XMZ34; XA40)

(44) Number of supraorbital bones: single (0); two (1); three or more (2). (Modified from GS14; XGC17; XM34; XMZ34; XA41)

Remarks: Based on personal observation, four supraorbital bones are present in *Luopingichthys bergi* from the Middle Triassic (Anisian) of Luoping, Yunnan.

(45) Multiple supraorbital bones arranged in more than one horizontal rows: absent (0); present (1).

Jaws and dentation

(46) Premaxilla: fused with antorbital, bearing sensory canal (0); present as distinct elements, lacking sensory canal (1); lost (2); fused with rostral, bearing sensory canal (3). (Modified from X30; XM36;

XMZ36; XA42)

Remarks: Personal reexaminations on the type specimens confirm that a pair of small, toothed premaxillae lacking sensory canal is present in *Platysiagum sinensis*.

(47) Premaxilla(e): present as a pair of elements (0); fused as a median element (1). (Modified from X30; XM36; XMZ36; XA43)

(48) Mobile premaxilla: absent (0); present (1). (Modified from GS24; XM37; XMZ37; XA44)

(49) Premaxilla immovably attached to braincase by means of a deep nasal process tightly sutured to frontals: absent (0); present (1). (G6; XW30; XGC45; XM38; XMZ38; XA45)

(50) Number of marginal teeth on premaxilla: three or more (0); one or two (1). (XA46)

(51) Foramen for olfactory nerve on premaxilla: absent (0); present (1). (Modified from G8; XW20; XGC46; XM39; XMZ39; XA47)

(52) Maxilla/preopercle contact: present (0); absent (1). (C15; XM40; XMZ40; XA48)

(53) Ventral portion of preopercle anteriorly extended, contacting maxilla anteriorly: absent (0); present (1). (Modified from XA49)

(54) Supramaxilla: absent (0); present (1). (Modified from GS22; XM41; XMZ41; XA50)

(55) Number of supramaxilla: single (0); two (1). (XZ28; XM42; XMZ42; XA51)

(56) Maxilla: present (0); absent (1). (XM43; XMZ43; XA52)

(57) Mobile maxilla in cheek: absent (0); present (1). (C16; XM44; XMZ44; XA53)

(58) Suborbital/maxilla contact: present (0); absent (1). (Modified from CA88; XGC56; XM45; XMZ45; XA54)

(59) Expanded dorsal lamina of maxilla: present (0); absent (1). (Modified from CA18; XA55)

(60) Depth of dorsal lamina of maxilla: no smaller than orbital length (0); smaller than orbital length (1). (XA56)

(61) Posterior margin of maxilla: slightly convex or straight (0); concave with a posterior maxillary notch (1). (GB62; XW46; XGC57; XM46; XMZ46; XA57)

(62) Oral margin of maxilla: concave or nearly straight (0); convex (1). (XA58)

(63) Posterior end of maxilla relative to orbit: well behind orbit (0); ending below (or nearly below) posterior orbital margin (1); ending nearly below orbital center or even anteriorly located (2). (XA59)

(64) Teeth on maxilla: present (0); much reduced or lost (1). (Modified from LZ11; XM47; XMZ47;

XA60)

(65) Distribution of teeth on maxilla: most of oral margin of maxilla (0); only anterior portion of oral margin of maxilla (1). (XA61)

(66) Extraordinarily long, fang-like teeth on both jaws: absent (0); present (1). (XA62)

(67) Molariform teeth on coronoid(s), prearticular and pterygoids: absent (0); present (1).

(68) Teeth on dentary: present (0); absent (1). (XM48; XMZ48; XA63)

(69) Quadratomandibular articulation: well behind orbit or below posterior orbit margin (0); nearly below the orbital center or even anteriorly located (1). (Modified from LZ2; X56; XGC58; XM49; XMZ49; XA64)

(70) Coronoid process: absent (0); present (1). (GS17; XGC23; XM50; XMZ50; XA65)

(71) Supra-angular bone in lower jaw: present (0); absent (1). (A67; XM51; XMZ51; XA66)

Palatoquadrate, Hyoid Arch, and Operculo-gular Series

(72) Vomers in adults: paired (0); fused (1). (XZS32; XM22; XMZ22; XA67)

(73) Suspensorium angle: acute (0); nearly vertical (1). (Modified from GSM29; XGC24; XM52; XMZ52; XA68)

(74) Hyomandibula with canal for hyoid branch of nerve VII: absent (0); present (1). (Modified from C22; XGC25; XM53; XMZ53; XA69)

(75) Dermohyal: present (0); absent (1). (Modified from GSM24; XM54; XMZ54; XA70)

(76) Postspiracle: absent (0); present (1). (XM55; XMZ55; XA71)

(77) Quadratojugal: plate-like (0); splint-like (1); much reduced or lost (2). (Modified from GSM26; C20; XGC26; XM56; XMZ56; XA72)

(78) Symplectic: absent (0); present (1). (Modified GSM13; XGC27; XM57; XMZ57; XA73)

(79) Symplectic involvement of jaw joint: absent (0); present (1). (GB61; G49; XW37; XGC28; XM58; XMZ58; XA74)

(80) Elongated posteroventral process of quadrate: absent (0); present (1). (Modified from GML24; XGC29; XM59; XMZ59; XA75)

(81) Number of hypobranchials: three (0); four (1). (G99; XW42; XGC60; XM60; XMZ60; XA76)

(82) Uncinate processes on epibranchials: absent (0); present (1). (C25; XGC30; XM61; XMZ61; XA77)

(83) Interopercle: absent (0); present (1). (GS18; XGC31; XM62; XMZ62; XA78)

(84) Two or more preopercular elements on each side of skull: absent (0), present (1). (XZ39; XM63;

XMZ63; XA79)

(85) Preopercle: present (0); absent (1). (XA80)

(86) Shape of preopercle: boomerang-shaped or irregular (0); crescent-shaped or L-shaped (1). (Modified from XZ40; XM64; XMZ64; XA81)

(87) Ventral end of preopercle located well above posterior end of oral margin of maxilla (when the ventral part of preopercle contacts maxilla): present (0); absent (1). (Modified from XA82)

(88) Opercle: present (0); absent (1). (XA83)

(89) Size of opercle: significantly larger than subopercle (0); nearly equal to or smaller than subopercle (1); much reduced (2). (Modified from XW43; XGC65; XM65; XMZ65; XA84)

(90) Suture between opercle and subopercle: slightly inclined or horizontal (0); greatly inclined (1). (XM66; XMZ66; XA85)

(91) Prominent anterodorsal process of subopercle: absent (0); present (1).

(92) Prominent anteroventral extension of subopercle: absent (0); present (1). (XA86)

(93) Number of branchiostegal rays: ten or more (0); seven to nine (1); four to six (2) two or three (3); single (4). (Modified from XA87)

(94) Gular bone(s): present (0); absent (1). (Modified from C11; XGC58; XM67; XMZ67; XA88)

Vertebrate and Caudal Skeleton

(95) Solid vertebral centra of adult-sized individuals: absent (0); present (1). (Modified from GB4; XGC68; XM68; XMZ68; XA89)

(96) Epipleural intermuscular bones: absent (0); present (1). (A103; XM69; XMZ69; XA90)

(97) Uroneural: absent (0); present (1). (XW56; XM70; XMZ70; XA91)

(98) Division of hypurals into dorsal and ventral groups (a gap between hypurals 2 and 3): absent (0); present (1). (XZS69; XM71; XMZ71; XA92)

Girdles and Fins

(99) Posttemporal: contacting extrascapular posteriorly (0); contacting extrascapular posterolaterally and separating this bone from contact with its counterpart (1); contacting extrascapular medially, and being incorporated into the skull roof (2); lost (3). (Modified from X27; XGC9; XM72; XMZ72; XA93)

(100) Width of posttemporals: broad, nearly as wide as extrascapular (0); relatively narrow, about half width of extrascapular series (1). (XA94)

Remarks: Based on personal observations, the posttemporals are relatively narrow in *Teffichthys*

madagascariensis. As such, the coding for this taxon is revised from ‘0’ to ‘1’.

(101) Clavicle: present as a broad plate (0); much reduced or lost (1). (Modified from GML37; XGC39; XM73; XMZ73; XA95)

(102) Supracleithrum relative to posterior margin of opercle in depth: supracleithrum nearly as deep as posterior margin of opercle (0); supracleithrum shorter than posterior margin of opercle (1); supracleithrum well deeper than posterior margin of opercle (2).

(103) Pectoral fins enlarged as wings: absent (0); present (1). (X66; XM74; XMZ74; XA96)

(104) Pelvic fins enlarged as auxiliary wings: absent (0); present (1). (X70; XM75; XMZ75; XA97)

(105) Number of dorsal and anal fin rays relative to radials: rays more numerous than radials (0); rays and radials nearly equal in number (1). (GSM31; XGC34; XM76; XMZ76; XA98)

Remarks: In *Cleithrolepis granulate*, the rays are more numerous than the radials in both the dorsal (19 radials corresponding to 27 rays) and anal (9 radials corresponding to 19 rays) fins (Wade, 1935). The similar condition is also present in *Polzbergia brochatus*, in which, the rays are also more numerous than the radials in the dorsal fin (Griffith, 1977).

(106) Dorsal and anal fin rays: segmented throughout the length (0); segmented distally (1). (XG62; XGC35; XM77; XMZ77; XA99)

(107) Origin of dorsal fin: nearly opposite to the origin of anal fin, well posterior to the origins of pelvic fins (0); between origins of anal and pelvic fins (1); anterior to origins of pelvic fins (2). (XA100)

(108) Caudal fin rays: terminate at caudal extremity of body axis (0); extend beyond termination of body axis (1). (Modified from GSM32; XGC36; XM78; XMZ78; XA101)

(109) Segmented procurrent rays in dorsal lobe of caudal fin: absent (0); present (1). (Modified from LZ19; XA102)

(110) Number of segmented procurrent rays in dorsal lobe of caudal fin: four or more (0); one to three (1).

(111) Number of principal caudal fin rays: 25 or more (0); 24 or less (1). (XA104)

(112) Principal rays of caudal fin ornamented with rounded ganoid tubercles: absent (0); present (1).

(113) Dense lepidotrichial segments of pectoral fin rays between innermost principle pectoral fin ray and body: absent (0); present (1). (X71; XGC37; XM79; XMZ79; XA105)

(114) Dense brush-like rays proximally articulating several stout segments at posterior portion of male anal fin: absent (0); present (1). (Modified from XZS74; XM80; XMZ80; XA106)

(115) Modification of male anal fin into unsegmented rays with tiny hooklets along anterior margin of

longest leading ray: absent (0); present (1). (XM81; XMZ81; XA107)

(116) Enlarged and posteriorly extended lateral scutes associate with anal fin: absent (0); present (1). (Modified from XM82; XMZ82; XA108)

(117) Anal fin relative to dorsal fin in size: anal fin smaller than or equal to dorsal fin (0); anal fin larger than dorsal fin (1). (XM83; XMZ83; XA109)

(118) Caudal fin: forked, lower lobe slightly shorter than or largely equal to upper lobe (0); forked, lower lobe longer than upper lobe (1); unforked (2). (Modified from X76; XGC74; XM84; XMZ84; XA110)

(119) Fringing fulcra on caudal fin: present (0); absent (1). (XM85; XMZ85; XA111)

(120) Fringing fulcra on pectoral fins: present (0); absent (1). (XM86; XMZ86; XA112)

Body shape and scales

(121) An apparent dorsal hump between head and dorsal fin: absent (0); present (1). (XW50; XA113)

(122) Scales: present (0); absent (1). (Modified from XGF67; XM87; XMZ87; XA114)

(123) Anterior flank scales: present (0); much reduced or absent (1). (Modified from XZS83; XM89; XMZ89; XA115)

(124) Lateral line scales: the lateral line scales as deep as or slightly deeper than those scales above and below (0); greatly deepened, 30% or more of the greatest depth of the body (1). (Modified from XZS83; XM89; XMZ89; XA116)

(125) Two horizontal rows of scales notably deepened in anterior flank region (lateral line scales notably deepened and nearly equal to the scales ventral to them): absent (0); present (1).

(126) Greatly deepened anterior flank scales corresponding to two or three horizontal rows of relatively shorter scales posteriorly: absent (0); present (1).

(127) Rhombic scales in anterior flank region: ornamented with ganoid ridges and tubercles (0); nearly smooth (1).

(128) Type of scales: ganoid of lepisosteoid type (0); elasmoid of amioid type (1); elasmoid of cycloid type (2). (A156; X80; XGC71; XM90; XMZ90; XA117)

(129) A posteriorly directed spine on dorsal ridge scale anterior to dorsal fin: absent (0); present (1). (GML36; LZ22; XW58; XA118)

(130) Posteriorly inclined scales in pectoral region: absent (0); present (1). (LZ24; XM92; XMZ92; XA119)

3. Data matrix

Moythomasia

00000000000-0-0----00000000000-00000000-000--000000000-00-00000000000000001000000000000

0000000000000000000000-0000000000000000000

Pteronisculus

00000000000-0-0----0000000001000100000100010--2-00-0000-00001000000000000001000000000000

0000000000000000000100-0000000000000000000

Boreosomus_piveteaui

00000000000-0-0----0000000101000001000100010--2-00-0000-0010100000000000000000?000000000

0200000000000000100000200-0000000000000000000

Acipenser

21020000111-0-0001000010110010?0?-10000---1002-00----0-1----------0-001-0010000000001-

-1--00310000200-0000100-0000000001000----000

Chondrosteus_acipenseroides

2?010000001-0-0???0?????1?0?1????-10000---0--2-00----0-1----------0-001-00100000??001-

-0200011000001000000200-0000000001001----000

Peipiaosteus_pani

2?01?0001?1-0-0???0?????1????????-10000---0--2-00----0-1----------0-001-00100000??001-

-020002100002?000000100-0000000001001----000

Australosomus

000000101210000???001101010010??00?0-010010--???0?0000-00101000000000000001000000010000

000000100000000010000011000000010110002001000

Brookvalia_gracilis

010020101210000???0?????????101000100010011002-00--000-000010000000000??0?002?????0000

0010004?????00020000010-0000000000000000000

Daedalichthys_higginsi

01001000121000???0?????????101000100010011002-00--000-00?010000000000??0?002?????0000

0010003?????00020000010-0000000000000000000

Redfieldius_gracilis

0100101010100 10???0?????????101000100010011002-00--000-001010000000000??0?002?????0000

0010004?????00020000011000000000000000001000

Helichthys_browni

010010101210010???0?????????1010001000100110 02-00--000-001010000000000??0?002?????0000

0010004?????00020000010-00000000000000001000

Platysiagum

000100010210000???0?????????10000011000-010--10?000000-001010000000000??1?00??????0000

001100100???00000000110-000000000000000(01)000

Helmolepis_cyphognathus

000100010010000???0?????????10000001000-010--??????000-001010000000000??1?00??????0000

000100100???00000000110-00000000000000001000

Cleithrolepidina

000100000211100???0?????????100000110111?0120??00-0000-00101001100010 0??1?00??????0000

001000??0???3-?100?011??0000000000100000000 1

Cleithrolepis

000?00000011100???0?????????100000110111001201000-0000-001010011000(01)00??1?00??????0

0000010000?0???3-??00001110000000000?1000000001

Polzbergia

?00?000??21?000???0?????????1???001101????1201000000?0-001010?1100000???1?00??????0?00

?0000???????3-0100000110000000000?0000000000

Plesiofuro

000100000211000???0?????????100000100111001201000000 00-001010000000000?1?002?????0000

001000200???00?00011110-10000000000000001000

Teffichthys

000100000211000---01110?100010000010011100120100000000-0010100000010 00??11002??00?0000

001000200???01?00011110-10000000000000001000

Meidiichthys_browni

000100000211000???0?????????10000010011100120100000000-00101000000?000??1?002??00?0000

00100020????00000011110-10000000000000001000

Perleidus_altolepis

000100000211000???0?????????10000010011100120100000000-001010000001000??1?002?????0000

00100020????01?0001111101000000000000000001000

Peltoperleidus_ducanensis

0001100?021-000???0?????????1000001001110001?01000?0010-0010100101000000??1?002?????0000

00100130????01?00011110-100000000000000101000

Peltoperleidus_macrodontus

0001-(02)000?1-000???0?????????100000100111001201000100010-001010010100000???1?002?????0

0000010013?????01?00011110-100000000000000101000

Pseudobeaconia_bracaccinii

010100000010000???0?????????10000011010-00110100000010-0010100101000000??1?002?????0000

00100130????01000011111110000000000?0000001010

Dipteronotus_olgiatii

00010000021100???0?????????1000001001110010??????010-0010100101?000??1???2?????0000

?01001??????0??000?1111?100000000?1000001010

Louwoichthys_illus

00010(02)000210000???0?????????1000001001110012010001001 0-001010010110000 0?1?002?????0

000001001300???0100001111101000000000000000001000

Ctenognathichthys

0001000002100000???0?????????1000001001110012010001 0010-001000010110000??1?002?????0000

0010013?????01?0001111100000000000000000001000

Luopingichthys_bergi

0001000002100000???0?????????100000100111001201000100 10-0010100101100000?1?002?????0000

001001300???0100001111100000000000000000001000

Colobodus_giganteus

0100000002110000???0?????????1000??11010-00121100000000-00-010000001000??1?002?????0000

0000100?????0??000?111110?000000000000000000

Colobodus_bassanii

0100000002110000???0?????????10000011010-001201000?0000-00-0100000010000?11002?????0000

000010000???01?000111111010000000000000000000

Colobodus_baii

0100000002110000???0?????????1000001?0?0-001?1100000000-00-0100000010000?11002?????0000

0000?0??????01?000?11110010000000000000000000

Crenilepis_sandbergeri

010000000211000???0?????????10000011010-00121100000000-00-0100000010000?11002?????0000

000010000???01?0001111100100000000000000000

Feroxichthys_yunnanensis

00000100021-000???0?????????1000-111010-00121110000000-00-010000101000??1?002?????0000

00001010????01?000?11110?1000000000000001000

Luganoia_lepidosteoides

10210100001-010???0?????????1000-110011000121310000000-000010020101010??11002?????0100

-01001??????01?10011211010000002110001011000

Luganoia_fortuna

10210100011-010???0?????????1000-1100110001??310000000-00001002010?010??1?002?????0000

-01001??????01?10011211010000002110001011000

Fuyuanperleidus_dengi

00010100021-000???0?????????100001100111001201000?0000-0010100001010000?1?002?????0000

00000020????01?10011111000000000110001011000

Peltopleurus_nitidus

00011(02)000110010???0?????????10000010011100120100000000-00101000010?000??1?012?????0

00010000030????01010011111110010110000001001000

Peltopleurus_tyrannos

00011(02)000010010???0?????????10000010011100110100000000-001010000101000??1?012?????0

00010000020????0??100?1111010010110000001001000

Peltopleurus_rugosus

000110000110010???0?????????10000010011100110??00?0000-00101000010?000??1?0?2?????0000

10000020????01?10011111010010100010001001000

Wushaichthys

001111-0021-010???0?????????10100010011000120100000000-00101000010?000??1?012?????0000

1000002000??11010011111010010100000001001000

Peripeltopleurus

001111-0021-010???0?????????10100010011000120100000000-00101000010?000??1?012?????0000

100000200???11?10011111010010101000001001000

Thoracopterus

001111-1121-010???0?????????10100010011???100100000000-00101000010?000??1?????????0000

100100000??011?111111110101???01110001001000

Potanichthys

001111-0121-010???0?????1?00101000100110001201100000000-00101000000001000??1??12?000?0000

100100?00?0011?1111111101011010111001----000

Gigantopterus

001111-??21-0?0???0?????????10?0001?01??00120?????0000-001010?00?0?000??11????????0000

?00100??0??01??111111110?01?0??111001----000

Thoracopterus_magnificus

001111-01?1-010???0?????????10?00010011?00???110000000-00101000000?000??1???2?00??0000

100100000?0011?111111110101?0?0111011-----00

Thoracopterus_martinisi

0011-2-01?1-010???0?????????10100010011?00???110?0?000-00101000000?000??1??12?00??0000

100100??0?0011?11111110?01?0?0111011-----00

Venusichthys

000100000210000???0?????????100000100110110--100000000-00101011010011??1?102?????0100

100000310???010100111111000000000000001000000

Habroichthys

1001-2-0?21-010???0?????????10?00011010-??0--1000??1-0-011010021-0010???1?102??0??0001

-000004?0???01?10111111010001001000111001000

Caturus

10010001121100111 00?????1?00101100101010111211001011-100111-10000000100111021101?1001

-0001000000000?00011210-0000000000000000-100

Amia

100100011210001110010101100010110010100-110--1001011-100111-10000000010011102110111001

-000100000000100011210-10000002110000000-100

Lepisosteus

1001000112100011110001111000111100100110111101001011-100---------000110011001100110001

--0010310000011000110 10-1000001200000000-000

Semionotus

10010001121000111 10?????1?0011110010001011120100101 1-100111-0020100011001110110011100 1

-0001011000001110011110-1000000000000000-010

Leptolepis

100100011210000000111101101110000010011011110101000 1-110111-0100000001111110210101100 1

-0000000111100110011110-1000000000000000-200

Elops

100100011211000010 11?10111101000001001 0-111001010001-110111-0100000001111110210101100 1

-0000000111100100011210-1000000011000000-200

5. References to Supplementary Information

Arratia G. 1999. The monophyly of Teleostei and stem-group teleosts. Consensus and disagreements. In: Arratia G, Schultze H-P, eds. *Mesozoic fishes 2 – systematics and fossil record*. München: Verlag Dr. Friedrich Pfeil, 265–334.

Arratia G. 2013. Morphology, taxonomy, and phylogeny of Triassic pholidophorid fishes (Actinopterygii, Teleostei). *Society of Vertebrate Paleontology Memoir (Supplement to Journal of Vertebrate Paleontology)* **13:** 1–138.

Brough J. 1931. The Triassic fishes of the Karroo System and some general considerations on the bony fishes of the Triassic period. *Proceedings of the Zoological Society* **1931:** 235–296.

Bürgin T. 1992. Basal ray-finned fishes (Osteichthyes; Actinopterygii) from the Middle Triassic of Monte San Giorgio (Canton Tessin, Switzerland). *Schweizerische Palaontologische Abhandlungen* **114:** 1–164.

Cartanyà J, Fortuny J, Bolet A, Mutter RJ. 2015. *Colobodus giganteus* (Beltan, 1972) comb. nov. from the Upper Muschelkalk facies of Catalonia (NE Iberian Peninsula). *Neues Jahrbuch für Geologie und Paläontologie, Abhandlungen* **278:** 323–333.

Cavin L. 2010. Diversity of Mesozoic semionotiform fishes and the origin of gars (Lepisosteidae). *Naturwissenschaften* **97:** 1035–1040.

Cloutier R, Arratia G. 2004. Early diversification of actinopterygians; In: Arratia G, Wilson MVH, Cloutier R, eds. *Recent Advances in the Origin and Early Radiation of Vertebrates*. München: Verlag Dr. Friedrich Pfeil, 217–270.

Coates MI. 1999. Endocranial preservation of a Carboniferous actinopterygian from Lancashire, UK, and the interrelationships of primitive actinopterygians. *Philosophical Transactions of the Royal Society of London B* **354:** 435–462.

Forey PL. 1973. A revision of the elopiform fishes, fossil and Recent. *Bulletin of the British Museum (Natural History), Geology* **10(Suppl):** 1–222.

Gardiner BG. 1984. The relationships of the palaeoniscid fishes, a review based on new specimens of *Mimia* and *Moythomasia* from the Upper Devonian of Western Australia. *Bulletin of the British Museum (Natural History), Geology* **37:** 173-428.

Gardiner BG, Maisey JG, Littlewood DTJ. 1996. Interrelationships of basal neopterygians. In: Stiassney MLJ, Parenti LR, Johnson GD, eds. *Interrelationships of Fishes*. San Diego: Academic Press, 117–146.

Gardiner BG, Schaeffer B. 1989. Interrelationships of lower actinopterygian fishes. *Zoological Journal of the Linnean Society* **97:**135–187.

Gardiner BG, Schaeffer B, Masserie JA. 2005. A review of the lower actinopterygian phylogeny. *Zoological Journal of the Linnean Society* **144:** 511–525.

Geng B-H, Jin F, Wu F-X, Wang Q. 2012. New perleidid fishes from the Middle Triassic strata of Yunnan Province. *Geological Bulletin of China* **31:** 915–927.

Grande L, Bemis WE. 1998. A comprehensive phylogenetic study of amiid fishes (Amiidae) based on comparative skeletal anatomy: An empirical search for interconnected patterns of natural history. *Society of Vertebrate Paleontology Memoir (supplement to Journal of Vertebrate Paleontology)* **4:**1–690.

Grande L. 2010. An empirical synthetic pattern study of gars (Lepisosteiformes) and closely related species, based mostly on skeletal anatomy. The resurrection of Holostei. *Copeia* **10 (Supplement):** 1–871.

Griffith J. 1977. The Upper Triassic fishes from Polzberg bei Lunz, Austria. *Zoological Journal of the Linnean Society* **60:** 1–93.

Hilton EJ, Forey PL. 2009. Redescription of †*Chondrosteus acipenseroides* Egerton, 1858 (Acipenseriformes, †Chondrosteidae) from the lower Lias of Lyme Regis (Dorset, England), with comments on the early evolution of sturgeons and paddlefishes. *Journal of Systematic Palaeontology* **7:** 427–453.

Hilton EJ, Grande L, William EB. 2011. Skeletal anatomy of the shortnose sturgeon, *Acipenser brevirostrum* Lesueur, 1818, and the systematics of sturgeons (Acipenseriformes, Acipenseridae). *Fieldiana–Life Earth Sciences* **3:** 1–168

Hutchinson P. 1973a. *Pseudobeaconia*, a perleidiform fish from the Triassic Santa Clara Formation, Argentina. *Breviora* **398:**1–24.

Hutchinson P. 1973b. A revision of the redfieldiiform and perleidiform fishes from the Triassic of Bekker's Kraal (South Africa) and Brookvale (New South Wales). *Bulletin of the British Museum of Natural History (Geology)* **22:** 235–354.

Lehman JP. 1952. Etude complémentaire des possions de l'Eotrias de Madagascar. *Kungliga Svenska Vetenskapsakademiens Hangdlingar* **2:** 1–201.

Lehman JP. 1979. Note sur les Poissons du Trias de Lunz. I. *Thoracopterus* Bronn. *Annalen des Naturhistorischen Museums in Wien* **82:** 53–66

Lombardo C. 2001. Actinopterygians from the Middle Triassic of northern Italy and Canton Ticino (Switzerland): anatomical descriptions and nomenclatural problems. *Rivista Italiana di Paleontologia e Stratigrafia* **107:** 345–369.

López-Arbarello A, Zavattieri AM. 2008. Systematic revision of *Pseudobeaconia* Bordas, 1944, and *Mendocinichthys* Whitley, 1953 (Actinopterygii: 'Perleidiformes') from the Triassic of Argentina. *Palaeontology* **51:** 1025–1052.

López-Arbarello A. 2012. Phylogenetic interrelationships of ginglymodian fishes (Actinopterygii: Neopterygii). *PLoS ONE* **7:** e39370.

Marramà G, Lombardo C, Tintori A, Carnevale G. 2017. Redescription of '*Perleidus*' (Osteichthyes, Actinopterygii) from the Early Triassic of northwestern Madagascar. *Rivista Italiana di Paleontologia e Stratigrafia* **123:** 219–242.

Mutter RJ. 2002. Revision of the Triassic family Colobodontidae sensu Andersson, 1916 (emended) whith a tentative assessment of perleidiform interrelationships (Actinopterygii: Perleidiformes). Unpublished Ph.D. thesis, Universität Zürich, Switzerland.

Mutter RI. 2004. The "perleidiform" family colobodontidae: A review. In Arratia G, Tintori A, eds. *Mesozoic Fishes 3 – Systematics, Paleoenvironments and Biodiversity.* München: Verlag Dr. Friedrich Pfeil, 197–208.

Neuman AG, Mutter RJ. 2005. *Helmolepis cyphognathus*, sp. nov., a new platysiagid actinopterygian from the lower Triassic Sulphur Mountain Formation (British Columbia, Canada). *Canadian Journal of Earth Sciences* **42:** 25–36.

Nielsen E. 1942. Studies on Triassic fishes from East Greenland. I. *Glaucolepis* and *Boreosomus*. *Meddelser øm Grønland* **138:** 1–403.

Nielsen E. 1949. Studies on Triassic fishes from East Greenland. II. *Australosomus* and *Birgeria*. Meddelser øm Grønland **146:** 1–309.

Olsen PE, McCune AR. 1991. Morphology of the *Semionotus elegans* species group from the Early Jurassic part of the Newark Supergroup of eastern North America, with comments on the family Semionotidae (Pisces: Neopterygii). *Journal of Vertebrate Paleontology* **11:** 269–292.

Patterson C. 1975. The braincase of pholidophorid and leptolepid fishes, with a review of the actinopterygian braincase. *Philosophical Transactions of the Royal Society of London, Series B* **269:** 275–579.

Pinna MCC. 1996. Teleostean monophyly. In: Stiassny MLJ, Parenti LR, Johnson GD eds. *Interrelationships of Fishes*. San Diego: Academic Press, 147–162.

Schaeffer B, McDonald NG. 1978. Redfieldiid fishes from the Triassic-Liassic Newark Supergroup of eastern North America. *Bulletin of the American Museum of Natural History* **159:** 129–173.

Sun Z-Y, Lombardo C, Tintori A, Jiang D-Y, Hao W-C, Sun Y-L, Lin H-Q. 2012. *Fuyuanperleidus dengi* Geng et al. 2012 (Osteichthyes, Actinopterygii) from the Middle Triassic of Yunnan Province, South China. *Rivista Italiana di Paleontologia e Stratigrafia* **118:** 359–373.

Sun Z-Y, Tintori A, Jiang D-Y, Lombardo C, Rusconi M, Hao W-C, Sun Y-L. 2009. A new perleidiform (Actinopterygii, Osteichthyes) from the Middle Anisian (Middle Triassic) of Yunnan, South China. *Acta Geologica Sinica* **83:** 460–470.

Sun Z-Y, Tintori A, Lombardo C, Jiang D-Y, Hao W-C, Sun Y-L, Wu F-X, Rusconi M. 2008. A new species of the genus *Colobodus* Agassiz, 1844 (Osteichthyes, Actinopterygii) from the Pelsonian (Anisian, Middle Triassic) of Guizhou, South China. *Rivista Italiana di Paleontologia e Stratigrafia* **114:** 363–376.

Tintori A. 1990. *Dipteronotus olgiatii* n. sp. (Actinopterygii, Perleidiformes) from the Kalkschieferzone of Ca' del Frate (N. Italy). *Atti Ticinensi di Scienze della Terra* **33:** 191–197.

Tintori A, Sassi D. 1992. *Thoracopterus* Bronn (Osteichthyes: Actinopterygii): a gliding fish from the Upper Triassic of Europe. *Journal of Vertebrate Paleontology* **12:** 265–283.

Wade RT. 1935. The Triassic Fishes of Brookvale, New South Wales. British Museum (Natural History), London, 110 pp.

Wen W, Hu S-X, Zhang Q-Y, Benton MJ, Kriwet J, Chen Z-Q, Zhou C-Y, Xie T, Huang J-Y. 2019. A new species of *Platysiagum* from the Luoping Biota (Anisian, Middle Triassic, Yunnan, South China) reveals the relationship between Platysiagidae and Neopterygii. *Geological Magazine* **156:** 669–682.

Xu G-H. 2020a. A new stem-neopterygian fish from the Middle Triassic (Anisian) of Yunnan, China, with a reassessment of the relationships of early neopterygian clades. *Zoological Journal of the Linnean Society*. DOI: 10.1093/zoolinnean/zlaa053.

Xu G-H. 2020b. A new species of *Luganoia* (Luganoiidae, Neopterygii) from the Middle Triassic Xingyi Biota, Guizhou, China. *Vertebrata PalAsiatica*. DOI: 10.19615/j.cnki.1000-3118.200624.

Xu G-H, Gao K-Q. 2011. A new scanilepiform from the Lower Triassic of northern Gansu Province, China, and phylogenetic relationships of non-teleostean Actinopterygii. *Zoological Journal of the Linnean Society* **161:** 595–612.

Xu G-H, Gao K-Q, Coates MI. 2015a. Taxonomic revision of *Plesiofuro mingshuica* from the Lower Triassic of northern Gansu, China, and the relationships of early neopterygian clades. *Journal of Vertebrate Paleontology* **35:** e1001515.

Xu G-H, Gao K-Q, Finarelli JA. 2014. A revision of the Middle Triassic scanilepiform fish *Fukangichthys longidorsalis* from Xinjiang, China, with comments on the phylogeny of the Actinopteri. *Journal of Vertebrate Paleontology* **34:** 747–759.

Xu G-H, Ma X-Y. 2016. A Middle Triassic stem-neopterygian fish from China sheds new light on the peltopleuriform phylogeny and internal fertilization. *Science Bulletin* **61:** 1766–1774.

Xu G-H, Ma X-Y, Zhao L-J. 2018. A large peltopleurid fish from the Middle Triassic (Ladinian) of Yunnan and Guizhou, China. *Vertebrata PalAsiatica* **56:** 106–120.

Xu G-H, Wu F-X. 2012. A deep-bodied ginglymodian fish from the Middle Triassic of eastern Yunnan Province, China, and the phylogeny of lower neopterygians. *Chinese Science Bulletin* **57:**111−118.

Xu G-H, Zhao L-J. 2016. A Middle Triassic stem-neopterygian fish from China shows remarkable secondary sexual characteristics. *Science Bulletin* **61:** 338–344.

Xu G-H, Zhao L-J, Gao K-Q, Wu F-X. 2012. A new stem-neopterygian fish from the Middle Triassic of China shows the earliest over-water gliding strategy of the vertebrates. *Proceedings of the Royal Society B* **280:** 20122261.

Xu G-H, Zhao L-J, Shen C-C. 2015b. A Middle Triassic thoracopterid from China highlights the evolutionary origin of over-water gliding in early ray-finned fishes. *Biology Letters* **11:** 20140960. Doi:10.1098/rsbl.2014.0960.

Zhou Z-H. 1992. Review on *Peipiaosteus* based on new material of *P. pani. Vertebrata PalAsiatica* **30:** 85–101.
